# An enhanced opposition-based African vulture optimizer for solving engineering design problems and global optimization

**DOI:** 10.1038/s41598-025-16630-0

**Published:** 2025-09-26

**Authors:** Henry Blankson, Vanisree Chandran, Himadri Lala, Prabhujit Mohapatra

**Affiliations:** 1 Department of Mathematics, SAS, VIT University, Vellore, Tamil Nadu 632014 India; 2 School of Electrical Engineering, VIT University, Vellore, Tamil Nadu 632014 India; 3 Department of CSE, SRM University-AP, Guntur, Andhra Pradesh India

**Keywords:** African vulture optimizer, Enhanced opposition-based learning, Metaheuristic, Random opposition-based learning, Applied mathematics, Computational science

## Abstract

By combining opposition-based learning techniques with conventional African Vulture Optimization (AVO), this study offers a notable improvement in the handling of optimization problems. Despite the limitations of AVO, such as issues involving extremely rough search spaces, more iterations or function evaluations are necessary. To overcome this limitation, our proposed paper, an enhanced opposition-based learning (EOBL), speeds up the convergence and, at the same time, assists the algorithm in escaping local optima. A combination of this new technique with AVO, the Enhanced Opposition-based African Vulture Optimizer (EOBAVO), is proposed. The performance of the suggested EOBAVO was evaluated through experiments using the CEC2005 and CEC2022 benchmark functions in addition to seven engineering challenges. Furthermore, statistical analyses, including the t-test and Wilcoxon rank-sum test, were conducted, and they demonstrated that the proposed EOBAVO surpasses several of the leading algorithms currently in use. The results indicate that the proposed approach can be regarded as a competent and efficient solution for complex optimization challenges.

## Introduction

Metaheuristic Algorithms (MA) are sophisticated optimization strategies that draw inspiration from natural processes and are intended to address challenging issues that conventional approaches find difficult to handle. To find the best answers, these algorithms, including simulated annealing, particle swarm optimization, and genetic algorithms, explore enormous search spaces. They are suitable in various domains, including engineering and finance, owing to their versatility and adaptability. These methods are widely acknowledged as global search algorithms that are stochastic, flexible, straightforward, and derivative free^1^. Their versatility and ease of use have made it possible to employ them to address a wide range of challenging business and science issues. Metaheuristic algorithms can be extensively categorized into two classes: Those that are entirely based on natural phenomena and those that are inspired by biological processes seen in nature. Swarm intelligence is another name for the biological processes found in nature. The term “swarm intelligence” was used by^2^ to refer to cellular robotic systems. Every swarm member controls its behavior and is separated from the others. In this case, the agent serves a different approach to the current issue. Instead of being present in every member, intelligence manifests in the swarm as a whole. As they change during the program, the agents of the problem (or solutions) are erratic^3^. Many metaheuristics have attracted the interest of multiple scholars and have received numerous citations over the last two decades. Table 1 lists some of the well-known algorithms.

**Table 1:** Types of metaheuristic algorithms.

Natural phenomenon	Biological processes or swarm intelligence
1. Simulated annealing (SA)^18^	1. Genetic algorithm (GA)^19^
2. Tabu search (TS)^20^	2. Particle swarm optimization (PSO)^4^
3. Harmony search (HS)^21^	3. Evolutionary strategy (ES)^22^
4. Covariance matrix adaptation of evolution strategy (CMAES)^23^	4. Evolutionary programming (EP)^24^
5. Big bang-big crunch (BBBC)^25^	5. Ant colony optimization (ACO)^26^
6. Biogeography-based optimization (BBO)^27^	6. Artificial bee colony (ABC)^5^
7. Gravitational search algorithm (GSA)^28^	7. Hunger games search (HGS)^29^
8. Teaching learning-based optimization (TLBO)^10^	8. Mountain gazelle optimizer (MGO)^30^
9. Black hole algorithm (BHA)^31^	9. Differential evolution (DE)^6^
10. Linear successful history-based adaptive de variants (LSHADE)^32^	10. Cuckoo search (CS)^7^
11. Sine cosine algorithm (SCA)^33^	11. Genetic programming (GP)^34^
12. Multi-verse optimizer (MVO)^35^	12. Grey wolf optimizer (GWO)^8^
13. Farmland fertility algorithm (FFA)^11^	13. Moth-flame optimization (MFO)^36^
14. Equilibrium optimizer (EO)^37^	14. Ant lion optimizer (ALO)^38^
15. Young double-slit experiment optimizer(YDSE)^39^	15. Whale optimization algorithm (WOA)^9^,
16. Cosine swarm algorithm (CSA)^40^	16. Salp swarm algorithm (SSA)^12^
	17. Aquila optimizer (AO)^41^
	18. African vulture optimization (AVO)^16^,
	19.Modified whale optimization algorithm (MWOA)^42^,
	20. White shark optimizer (WSO)^43^
	21. American zebra optimization algorithm (AZOA)^44^

Although most of the previously stated algorithms have been applied to many optimization problems, they have been used by metaheuristic algorithms and show that slow convergence or falling into local optima remains a common issue. For instance, being trapped in local optima and experiencing premature convergence are two problems associated with the PSO method^4^. The ABC algorithm^5^ features a poor balance between exploitation and exploration and a slow rate of convergence. The Differential Evolution (DE) methodology exhibits specific shortcomings, including a protracted convergence rate and population stagnation^6^. The Cuckoo Search^7^ algorithm has numerous drawbacks, including inadequate search velocity and diminished convergence precision. The Grey Wolf Optimizer (GWO) has drawbacks, including a tendency to become trapped in local optima and a slow rate of convergence during the final stages of the search process^8^. Some of the shortcomings of the WOA^9^ include poor accuracy, sluggish convergence, and susceptibility to local optima. The convergence rate of TLBO is its primary drawback, and it becomes significantly more problematic when handling high-dimensional problems^10^. The Fast Fourier Algorithm (FFA) presents several shortcomings, including a tendency for slow convergence and uneven distribution within intensification (exploitation) and diversification (exploration) efforts^11^. Salp Swarm Algorithm (SSA) also has challenges related to population variety and being trapped in locally optimal solutions^12^. Some of the drawbacks of GSA include complex objective functions, which may have high computational requirements, intricate procedures, a large number of control parameters, and poor convergence^13^. Among the shortcomings of the Sea Horse Optimizer (SHO) are its sluggish rate of convergence and propensity to become stuck in local optima^14^. The low variety and unequal use of exploitation and exploration are problems with Golden Jackal Optimization (GJO)^15^. Similarly^16^, presented the drawbacks of the African Vulture Optimization Algorithm (AVO) as the addition of exploitation capability to the exploration phase to speed up convergence, and the random strategy that determines the changeover between the phases of exploration and exploitation, which impacts balance. This idea causes local optimum trapping and affects the global search of the region. To overcome the aforementioned restrictions, new optimization techniques are required. Furthermore^17^, proposed the no-free-lunch theorem (NFL), where no algorithm can be successful in every optimization problem. Owing to the above limitations, there is always room to create new metaheuristic algorithms or modify existing ones to resolve challenging optimization problems in a variety of domains. Table 1 lists the types of metaheuristic algorithm.

In their quest to enhance the existing algorithms^45^, introduced an improved SCMSSA aimed at enhancing both the optimization precision and efficiency. It also assesses SCMSSA in comparison with alternative algorithms through the application of six distinct test functions. The SVR-SCMSSA model demonstrated a 95% accuracy in predicting CO2 emissions, providing valuable insights into the primary factors contributing to CO2 emissions. In another study^46^, presented a Modified EDO (MEDO) that integrates EDO with SSA and QI. An adaptive p-best mutation technique was employed to avoid local optimum pitfalls. In addition, a phasor operator is utilized to improve the diversity within the algorithm^47^ also introduced the Salp Navigation and Competitive based Parrot Optimizer (SNCPO). This involves a hybrid algorithm combining Competitive Swarm Optimization and Salp Swarm Algorithm. Their findings indicate that SNCPO consistently surpasses current leading algorithms, attaining enhanced convergence rates, solution quality, and robustness, while successfully evading local optima. Importantly, SNCPO shows significant adaptability to various optimization environments, underscoring its applicability in practical engineering and machine learning scenarios. The exploitation phase was based on an exponential distribution model.

The following is a summary of the main contributions of this research.

i. To enhance the exploration and exploitation of conventional AVO, a recently developed method called Enhanced Opposition-based Learning (EOBL) is suggested and integrated into AVO to create an Enhanced Opposition-based African Vulture Optimizer (EOBAVO) algorithm.
ii. Statistical tests validated the performance of the proposed methodology and compared it with eight of the best algorithms. Moreover, EOBAVO was assessed based on engineering challenges to show that it can solve real-life engineering problems.
iii. The efficacy and toughness of EOBAVO were confirmed using the CEC2005 benchmark’s 23 test functions, which had both low and high dimensions.
iv. Exploration–Exploitation and Diversity Analyses show that EOBAVO effectively transitions from exploration to exploitation and converges well on most functions and enhanced convergence speed by reducing population diversity and promoting exploitation across benchmarks.
v. Additionally, EOBAVO was tested on some engineering challenges to show that real-life engineering complications can be resolved.

The rest of the paper is structured as follows: Section “Preliminaries” offers a literature review on AVO, a brief justification and mathematical modeling of the traditional AVO technique, and an outline of the fundamentals of ROBL and OBL techniques. Section “The Proposed EOBAVO” describes the EOBAVO algorithm and the fundamental idea of IOBL. Section “Numerical experiments and results analysis” presents numerical tests and analysis of the results. Section “Performance of EOBAVO on practical engineering problems” shows how the EOBAVO algorithm can be used to solve actual engineering challenges, and Section “Conclusion” summarizes the research and suggests areas that require further investigation.

## Preliminaries

### Literature review on AVO

The African Vulture Optimization (AVO), a novel MA created by Abdollahzadeh et al. in 2022, is an intriguing substitute for global optimization^48^. Inspired by the hunting habits of African vultures, AVO consists of two primary steps: Finding prey and attacking it^16^. AVO is more effective on a few benchmark functions when compared to the sophisticated metaheuristic algorithms previously discussed. AVO is currently employed to resolve a range of challenges in the field of engineering optimization, as well as in numerous other areas of study. For example, to identify the ideal parameters of a solid oxide fuel cell (SOFC) steady-state model^49^, used various swarm intelligence methods for SOFC parameter estimation. The findings demonstrate that AVO is capable of producing precise characteristic curves for voltage and current using the 8-population intelligent optimization algorithm to solve five mechanical part design problems^50^, showed that AVO had the quickest solution time and ensured reasonable performance. The analysis of the merits and limitations of the established MAs, coupled with efforts to enhance their functionality through the introduction of new or modified mechanisms, presents an emerging research challenge. Recent developments in machine learning, termed as opposition-based learning (OBL), are perceived as an effective strategy for boosting the efficiency of these algorithms. Motivated by the opposite link between items^51^, first presented the concept of opposition in 2005. Over the past ten years, scholars have paid considerable attention to this topic. The concept of OBL has been used to enhance the functionality of a range of soft computing algorithms, such as artificial neural networks, fuzzy systems, optimization techniques, and reinforcement learning. The integration of the OBL methodology with additional bio-inspired optimization techniques yields shorter estimated distances to the global optimum. For instance, OBL is used in the DE optimization algorithm to generate new offspring when the species changes^52^. In addition, with the introduction of OBL to the Grasshopper Optimization Algorithm^53^, it was possible to swiftly reach an optimal point, and the exploration region was fully explored.

**Fig. 1:**
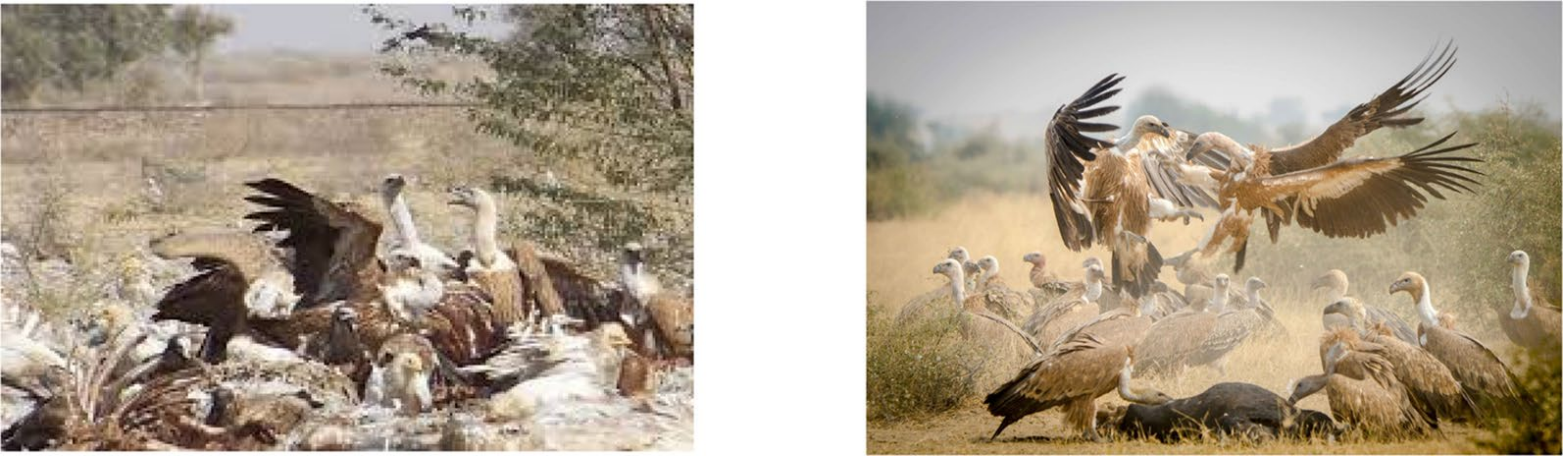
Hunting behaviour of vultures.

Nature-inspired optimization techniques, such as AVO, have been created based on the behaviors and feeding habits of African vultures. The ability of vultures to locate carrions across great distances and their remarkable scavenging skills are well-recognized. (Sharp Vision and Communication). Figure 1 shows the hunting behavior of the vultures.

They can also collaborate to identify the best answers by drawing on the insights of others (Social Cooperation) and depending on their surroundings, with their dynamic role adaptation, vultures alternate between exploring (looking for food) and exploitation (eating)^54^. To address challenging optimization problems, the AVO algorithm attempts to imitate these characteristics. AVO has shown notable applicability in several domains such as data analysis, power optimization, and control systems. It works well for complicated optimization problems because of its simplicity and minimal processing requirements^55^.

The nature-inspired metaheuristic algorithm known as AVO was created by^16^ and was inspired by the way vultures, which hunt birds, search for food. Vultures in their natural habitat behave differently from other birds in the scavenging process, continuously traveling great distances in a revolving fight style. Vultures, when searching for food, also look for other members of their kind that have found food. Vultures from several species occasionally gather from similar food sources. It is very rare to find weaker vultures around stronger ones, since they fight them for food.

The AVO principle, as described in^54^, imitates the population of vultures. Based on the fitness value, the initial population of N vultures in the AVO algorithm was divided into three groups. The population’s best solution is found in the first category. The second-best vultures are in the second team, and the rest are in the third group. Each vulture group plays a unique role in a food hunt. The most prevalent and dominant vultures, that is, the finest solutions, were thought to be the best in AVO. However, the worst solutions are the most hungry and feeblest vultures in the population. To discover the optimal alternative, vultures in AVO try to get closer to strong vultures and stay away from weak vultures. Based on the core concept of vultures and the theoretical frameworks established for the modeling of artificial vulture populations, the development of the AVO algorithm occurred in four phases,

### Phase 1: categorizing the population according to the best vulture

To determine the leading solutions, the fitness value of each solution is calculated following the creation of the initial population. The best solution was determined by selecting the best vulture for the first category, and the second-best solution was determined by selecting the best vulture for the second category. Equation (1) determines the probability of successfully guiding the vulture towards the optimal solution within the two categories. This probability is subsequently used to move the remaining responses towards the best solutions identified in both the first and second categories.

1 $$\begin{array}{*{20}c} {R\left( i \right) = \left\{ {\begin{array}{*{20}l} {BestVulture_{1} } \hfill & {if} \hfill & {p_{i} = L_{1} } \hfill \\ {BestVulture_{2} } \hfill & {if} \hfill & {p_{i} = L_{2} } \hfill \\ \end{array} } \right.} \\ \end{array}$$

**Fig. 2:**
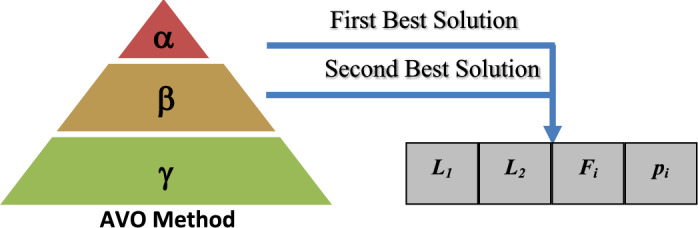
The connection between the AVO.

In the first group, the best vulture is designated *as BestVulture*_*1*_, and the second-best vulture in the second category is designated *as BestVulture*_*2*_. The sum of random numbers L_1_ and L_2_, which ranges from 0 to 1, is 1. *pi* can be calculated using Eq. (2) and the roulette-wheel method.

2 $$\begin{array}{*{20}c} {p_{i} = \frac{{F_{i} }}{{\mathop \sum \nolimits_{i = 1}^{n} F_{i} }}} \\ \end{array}$$

where *Fi* represents the fitness value for the groups and the total number of vultures in the two categories is represented by n. In conclusion, Fig. 2 illustrates the relationships between the vultures, where $\boldsymbol{\alpha }$ represents the first group, ${\varvec{\beta}}$**,** the second group and ${\varvec{\gamma}}$, the third group of vultures. The target vulture was acquired using the pertinent characteristics.

### Phase 2: vultures going without food (starvation)

Because they are powerful animals, vultures can fly great distances to find food when they are not famished. On the other hand, when they are hungry, they become hostile and unable to fly very far, forcing them to approach stronger vultures in their quest for food. Equation (3) was used to mathematically model this phenomenon:

3 $$\begin{array}{*{20}c} {t = h \times \left( {sin^{k} \left( {\frac{\pi }{2} \times \frac{itr\left( i \right)}{{itr_{max} }}} \right) + cos\left( {\frac{\pi }{2} \times \frac{itr\left( i \right)}{{itr_{max} }}} \right) - 1} \right)} \\ \end{array}$$

The Hunger rate Eq. (4) is used to determine the vultures’ appetite to determine when the exploration phase ends and the exploitation phase begins.

4 $$\begin{array}{*{20}c} {F = \left( {2 \times rand_{1} + 1} \right) \times Z \times \left( {1 - \frac{itr\left( i \right)}{{itr_{max} }}} \right) + t} \\ \end{array}$$

where variables h, Z, and *rand*_*1*_ are random integers drawn from [− 2, 3], [− 1, 1], and [0, 1] intervals, respectively; F represents the degree of hunger; and *itr*_*max*_ and *itr*(i) indicate the maximum and current iterations, respectively. The probability of initiating the exploration process during the final stages of optimization is positively correlated with an increase in the value of k, whereas a reduction in the parameter leads to a lower likelihood of entering this exploration phase. This fixed value of k indicates that the optimization process impedes the exploration and exploitation stages. The formula for rate F reveals that the operational rate of the vultures decreases as the number of iterations increases. In particular, if F is greater than 1, vultures will continue to explore and seek food in diverse areas. However, if F is less than or equal to 1, the vultures enter the exploitation phase, where they search for food closer to the existing solution.

### Phase 3: exploration

Vultures can easily discover food and carcasses in the wild with the aid of their excellent vision. Vultures take time to assess their surroundings before starting a protracted food battle. Vultures in AVO can investigate several random sites using one of two methods; the parameter *p*_*i*_ is used to select the strategy. Which of the two strategies will be employed is determined by this parameter value, which is predefined in the range between 0 and 1 before the search procedure begins. Equation (5) can be used to depict the exploration phase of the vultures.

5 $$\begin{array}{*{20}c} {X_{t + 1} = \left\{ {\begin{array}{*{20}l} {R_{t} - D_{t} \times F_{t} ,} \hfill & {p_{1} \ge rand_{p1}^{t} } \hfill \\ {R_{t} - F_{t} + rand_{i2}^{t} \times \left( {\left( {ub - lb} \right) \times rand_{i3}^{t} } \right) + lb,} \hfill & {p_{1} < rand_{p1}^{t} } \hfill \\ \end{array} } \right.} \\ \end{array}$$

where $X_{t + 1}$ represents the vulture position in subsequent iterations, one of the best vultures, Rt, is determined by Eq. (1) and $rand_{p1}^{t}$, $rand_{p2}^{t}$, and $rand_{p3}^{t}$ are raom values between 0 and 1. *F*_*t*_ is determined by Eq. (4); the lower and upper bounds are also represented by *lb* and *ub,* respectively; and finally, *D*_*t*_ denotes the vulture’s distance from the current optimal vulture, which is determined by Eq. (6).

6 $$\begin{array}{*{20}c} {D_{t} = \left| {C \times R_{t} - X_{t} } \right|} \\ \end{array}$$

where C represents a random integer distributed uniformly between 0 and 2, and the vulture position currently being used in the iteration is represented by *X*_*t*_.

### Phase 4: exploitation’s first stage

The exploitation phase in AVO is grouped into two main stages. The first stage starts when F has a value ranging between 0.5 and 1. Two distinct tactics are employed during this phase: Siege fighting and rotating combat. The predetermined random value p_2_$\in$[0, 1] is utilized to choose which approach to use. The Siege-fight (food competition) approach is used if the generated number is greater than p_2_, which is the initial random variable *rand*_*p*_*2* created randomly in the interval [0,1]. The rotating combat tactic is used elsewhere^56^.

- *Competing for food* When F falls between 0.5 and 1, the vultures are deemed to be active and full. As a result, the weak vultures gather and attempt to attack the vital ones to get food, while the stronger vultures are unwilling to share their food. Equation (7) and (8) shows the modeled behavior to update the location of the vulture.

7 $$\begin{array}{*{20}c} {X_{t + 1} = D_{t} \times \left( {F_{t} + rand_{4}^{t} } \right) - d_{t} } \\ \end{array}$$

8 $$\begin{array}{*{20}c} {d_{t} = R_{t} - X_{t} } \\ \end{array}$$

- *Rotating flight strategy* Vultures that are motivated and stuffed will hover at high altitudes and compete for food. A spiral model is used by AVO to mimic this behavior. The expression for rotating flight strategy can be described using Eqs. (9)–(11). The positional update equation pertinent to the rotational flight dynamics of vultures is given by Eq. (9).
  9 $$\begin{array}{*{20}c} {X_{t + 1} = R_{t} - \left( {S_{1}^{t} + S_{2}^{t} } \right)} \\ \end{array}$$
  10 $$\begin{array}{*{20}c} {S_{1}^{t} = R_{t} \times \left( {\frac{{rand_{5} \times X_{t} }}{2\pi }} \right) \times cos\left( {X_{t} } \right)} \\ \end{array}$$
  11 $$\begin{array}{*{20}c} {S_{2}^{t} = R_{t} \times \left( {\frac{{rand_{5} \times X_{t} }}{2\pi }} \right) \times sin\left( {X_{t} } \right)} \\ \end{array}$$

All rand values in this phase are equally distributed and fall between 0 and 1.

### Phase 5: the final stage of exploitation

The second and final stage of exploitation is when the movements of the vultures gather several vulture species over the food supply. During this stage, the methods of siege or aggressive conflict, struggling for food, are carried out. If the value of F is below 0.5, the algorithm advances to this stage. To begin this step, labelled rand *P*_*3*_, is produced with bounds of [0, 1]. If *rand*_*P3*_ ≥ *P*_*3*_, the vultures display the aggregation behavior. However, if the generated number is greater than *P*_*3*,_ yet less than 0, the vultures will adopt an attack behavior.

- *Aggregation behavior* Vultures digest a lot of food when AVO reaches its last stage. Vultures will gather in large numbers and act competitively wherever food is available. Currently, Eq. (12) is used to calculate the formula for updating the vultures’ position.

12 $$\begin{array}{*{20}c} {X_{t + 1} = \frac{{A_{1}^{t} + A_{2}^{t} }}{2}} \\ \end{array}$$

13 $$\begin{array}{*{20}c} {A_{1}^{t} = BestVulture_{1}^{t} - \frac{{BestVulture_{1}^{t} \times X_{t} }}{{BestVulture_{1}^{t} - \left( {X_{t} } \right)^{2} }} \times F_{t} } \\ \end{array}$$

14 $$\begin{array}{*{20}c} {A_{2}^{t} = BestVulture_{2}^{t} - \frac{{BestVulture_{2}^{t} \times X_{t} }}{{BestVulture_{2}^{t} - \left( {X_{t} } \right)^{2} }} \times F_{t} } \\ \end{array}$$

- *Attack behavior* In a similar manner to when AVO is almost finished, the vultures will approach the best vulture to take the leftover food. Mathematically, the following expression for updating the position of the vultures is given by Eqs. (15), (16), and (17).
  15 $$\begin{array}{*{20}c} {X_{t + 1} = R_{t} - \left| {d_{t} } \right| \times F_{t} \times Levy\left( {dim} \right)} \\ \end{array}$$
  16 $$\begin{array}{*{20}c} {Levy\left( {dim} \right) = 0.01 \times \frac{{r_{1} \times \sigma }}{{\left| {r_{2} } \right|^{{\frac{1}{\delta }}} }}} \\ \end{array}$$
  17 $$\begin{array}{*{20}c} {\sigma = \left( {\frac{{{\Gamma }\left( {1 + \delta } \right) \times sin\left( {\frac{\pi \delta }{2}} \right)}}{{{\Gamma }\left( {1 + \delta 2} \right) \times \delta \times 2^{{\left( {\frac{\delta - 1}{2}} \right)}} }}} \right)^{{\frac{1}{\delta }}} } \\ \end{array}$$
  where δ = 1.5 is a constant, and *r1* and *r*_*2*_ are the random values that are uniformly distributed within [0, 1], and dim represents the problem dimension.

**Fig. 3:**
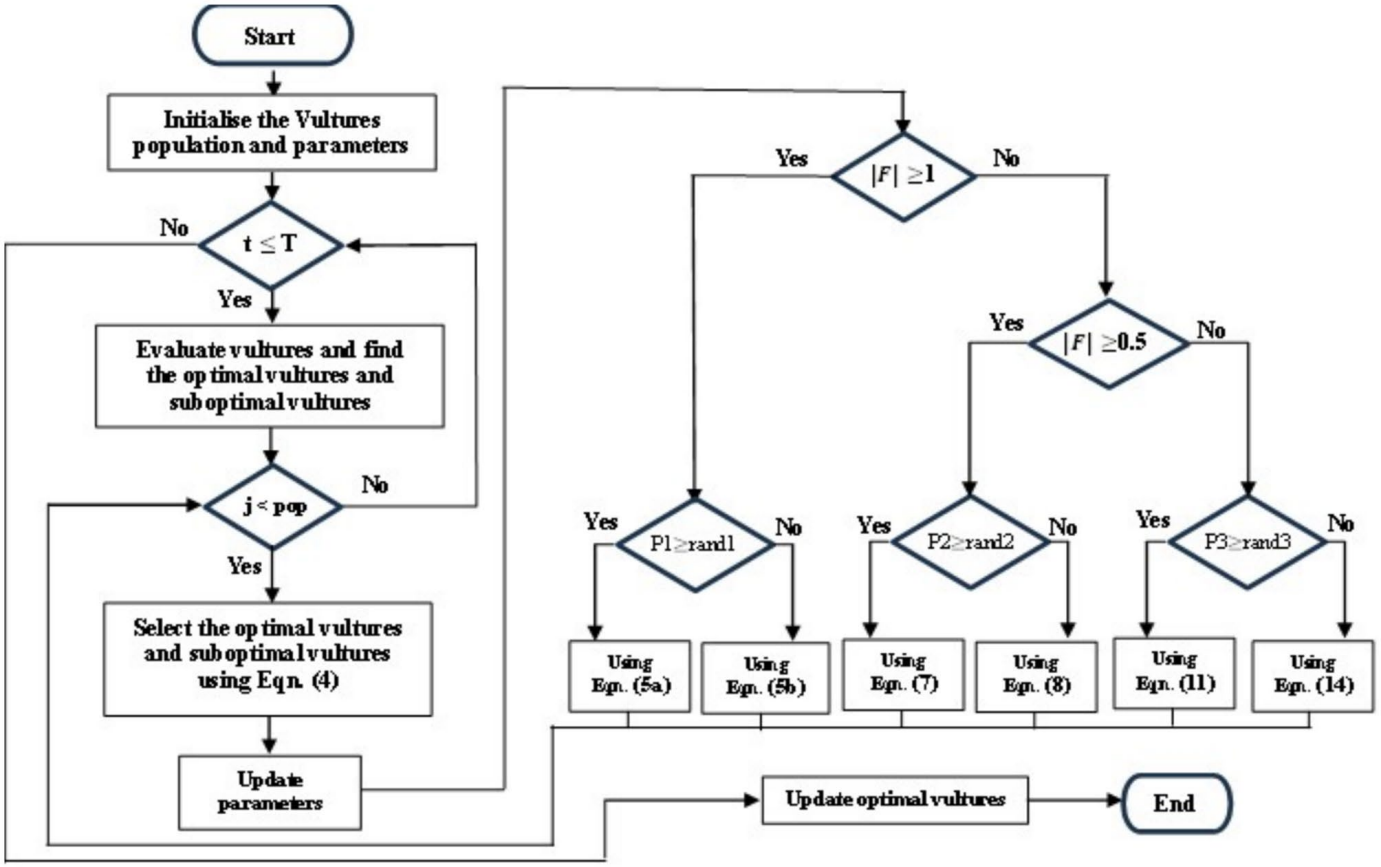
Flowchart of the African vulture optimization algorithm.

Figure 3 depicts the African vulture optimization algorithm’s solution procedure.

### The opposition-based learning (OBL)

To enhance the efficiency of MAs in solving complex optimization challenges, Tizhoosh introduced the idea of Opposition-based learning (OBL) in 2005^51^. The erratic, radial changes brought about by societal revolutions serve as the main driving force behind the OBL approach. MAs use OBL to address two major challenges in optimization, namely, premature convergence and slow exploration of the search space^51^. In other words, OBL serves as an effective strategy to enhance the performance of MAs by evaluating candidate solutions with their opposites^52^. This mechanism significantly improves search space exploration, increases the probability of locating global optima, and mitigates premature convergence. Within the AVO algorithm, the integration of OBL strengthens the balance between exploration and exploitation, accelerates convergence rates, and reduces the risk of stagnation in local optima when addressing complex, multimodal optimization problem^8,57^. The incorporation of OBL enables MAs to enhance population diversity while simultaneously sustaining a dynamic equilibrium between exploration (global search) and exploitation (local search refinement) during the optimization process. The improved exploration ability diminishes the chances of getting trapped in local minima and hastens the process of reaching global optima, especially in complex high-dimensional and multimodal environments^52^. Additionally, OBL promotes a more resilient search mechanism by methodically addressing stagnation, enabling the algorithm to break free from plateaus or misleading areas within the search domain^58^. The AVO algorithm significantly benefits from the integration of OBL approach, which enhances its search efficiency. This allows the agents, or vultures, to effectively navigate promising regions while preserving diversity to prevent premature convergence^8,58^. As a result, this leads to improved convergence rates, superior solution quality, and increases stability when addressing intricate, nonlinear, and multimodal optimization challenges.

Therefore, the integration OBL into MAs like AVO represents a significant advancement in the design of optimization strategies, offering a practical and theoretically grounded solution to the challenges of exploration–exploitation balance and robustness in complex search spaces. The OBL concept has been used in AVO successfully as the Opposition-based learning African Vulture Optimization Algorithm (OBAVO) to improve the exploitation potential of the original AVO’s search mechanism^59^. Additionally, the next subsections explain the opposite number idea.

#### The opposite number

For any random variable $V \in \left| {a, b} \right|$, the opposite number $\hat{V},$ can be found using the formula in Eq. (18) below.

18 $$\begin{array}{*{20}c} {\hat{V} = a + b - V } \\ \end{array}$$

where a and b represent the search space’s lower boundary and upper boundary, respectively. The population’s initial position is denoted by V. Equation (19) below can be used to generalize Eq. (13) into n-dimensional space:

19 $$\begin{array}{*{20}c} {{\hat{\text{V}}}_{{{\text{ij}}}} = {\text{a}}_{{{\text{ij}}}} + {\text{b}}_{{{\text{ij}}}} - {\text{V}}_{{{\text{ij}}}} \quad i = 1,2, \ldots N} \\ \end{array}$$

where the real vector $V \in R^{n}$ has an opposite value of $\hat{V} \in R^{n}$. Nevertheless, throughout any optimization process, the values V and $\hat{V}$ are analyzed. By comparing the objective functions, the best of these two outcomes is maintained, and the worst is removed. For example, *V* is saved if *F(V)* is less than $F\left( {\hat{V}} \right)$, otherwise if *F(V)* is greater than $F\left( {\hat{V}} \right)$, then we save $\hat{V}$.

**Fig. 4:**
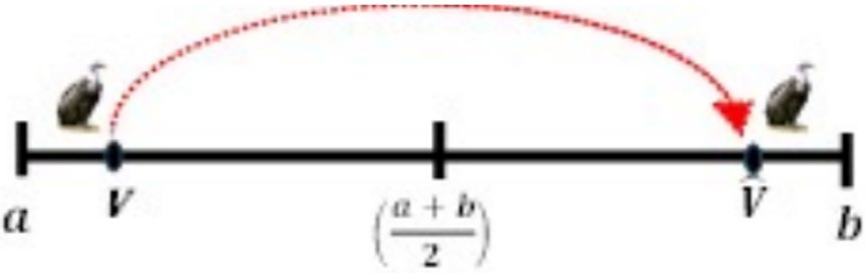
One-dimensional OBL mechanism space.

**Fig. 5:**
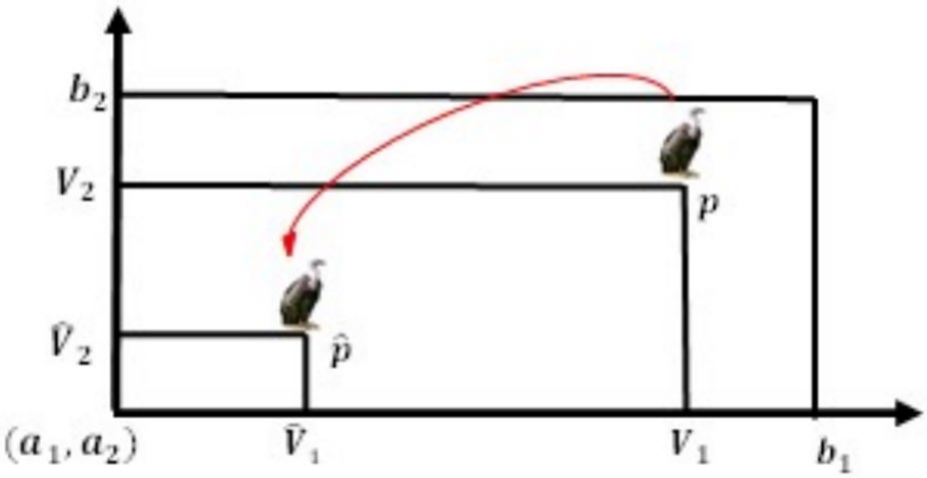
Two-dimensional OBL mechanism space.

**Fig. 6:**
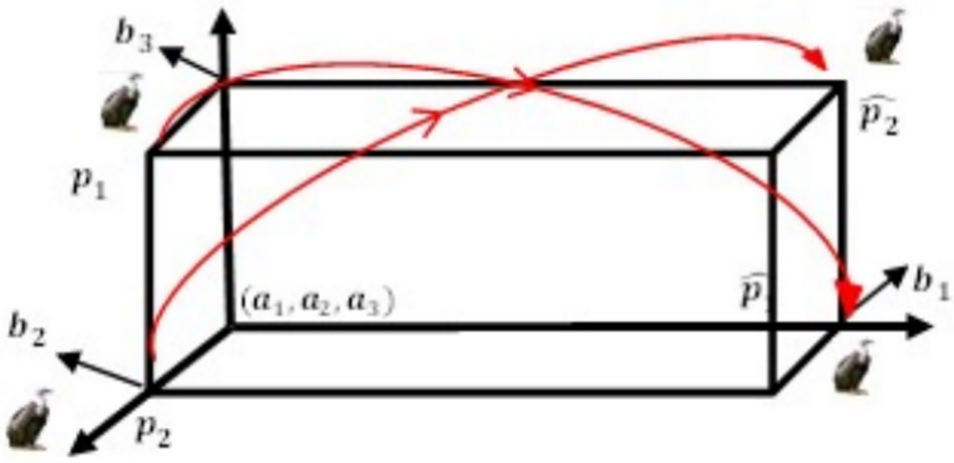
Three-dimensional OBL mechanism space.

V and its opposite $\hat{V}$, are shown in one, two, and three dimensions, respectively, in Figs. 4, 5, and 6

### ROBL

**Fig. 7:**
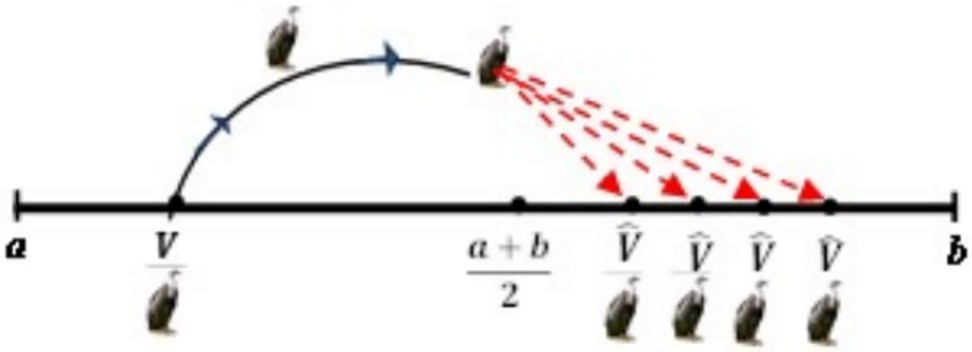
One-dimensional ROBL mechanism space.

**Fig. 8:**
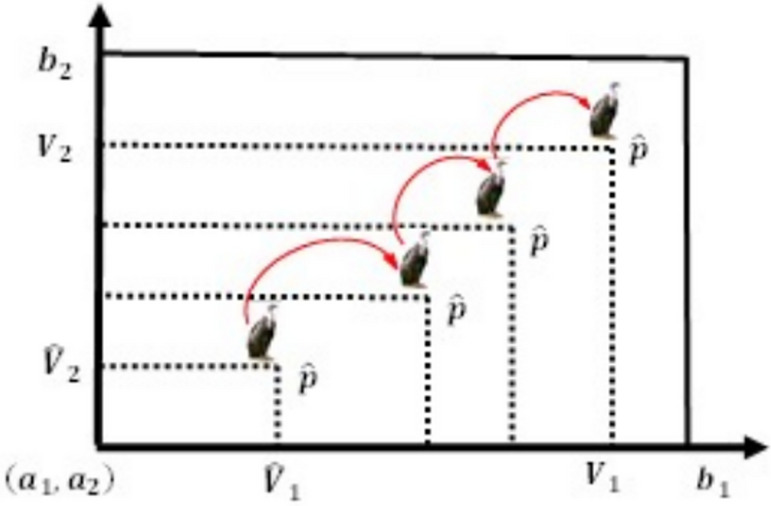
Two-dimensional ROBL mechanism space.

**Fig. 9:**
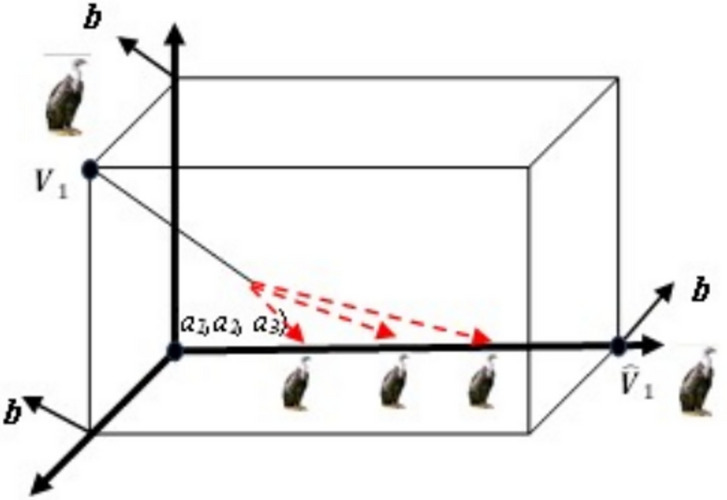
Three-dimensional ROBL mechanism space.

To help evade becoming trapped in local optima and also increase diversity, the Random Opposition-based Learning (ROBL) was created by^60^, a novel approach to OBL, in 2019. ROBL incorporates African Vulture Optimizer (AVO) as the Random Opposition-Based learning African Vulture Optimizer (ROBAVO). In contrast to Eq. (19), the opposite solution $\hat{V}_{ij}$, represented by Eq. (20), is random for investigation. It describes a novel OBL strategy known as Random Opposition-Based Learning (ROBL)

20 $$\begin{array}{*{20}c} {{\hat{\text{V}}}_{{{\text{ij}}}} = {\text{a}}_{{{\text{ij}}}} + {\text{b}}_{{{\text{ij}}}} - {\text{rand*V}}_{{{\text{ij}}}} ,\quad i = 1,2,3 \ldots N} \\ \end{array}$$

where *rand* is between 0 and 1, and ${a}_{ij}, {b}_{ij}$ represent the lower and upper limits of the i^th^ particle, respectively. Therefore, Eq. (20) can effectively increase the population’s diversity and also assist in avoiding local optima. The V and its opposing $\widehat{V}$ are shown in one, two, and three dimensions, respectively, in Figs. 7, 8, and 9.

## The proposed EOBAVO

### The enhanced opposition-based learning (EOBL)

The Enhanced Opposition-based Learning (EOBL) represents a groundbreaking pedagogical strategy designed to aid in the assessment of a candidate solution by concurrently evaluating the current response alongside its developed counter-solution. This technique can facilitate a faster convergence of the optimization algorithm by selecting the most appropriate correspondence between the current solutions being compared and their evolving opposite-based counterparts, which will be refined in later iterations.

This makes it possible for the first response to be the best fit solution, which is the guess or enhanced-opposite approximation that tackles the issue. The process begins with the closer of the two guesses. Every other solution in the present population can be implemented consistently using the same process.

**Fig. 10:**
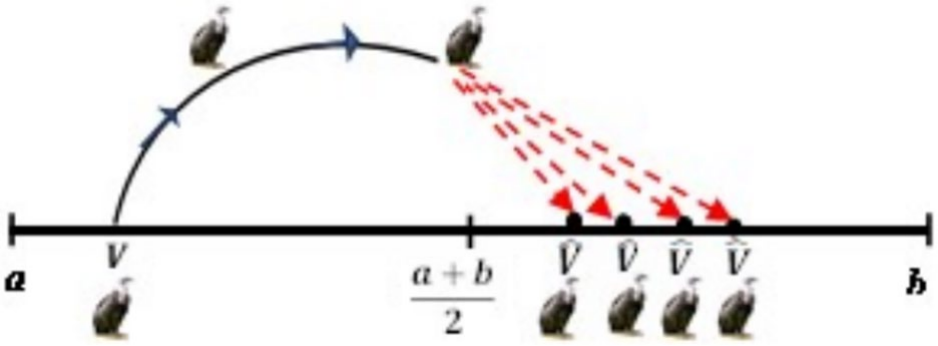
One-dimensional EOBL mechanism space.

**Fig. 11:**
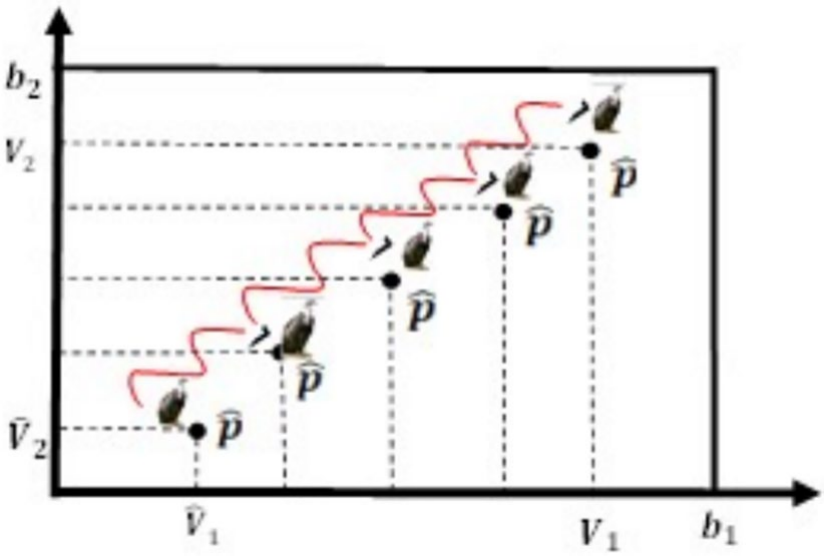
Two-dimensional EOBL mechanism space.

**Fig. 12:**
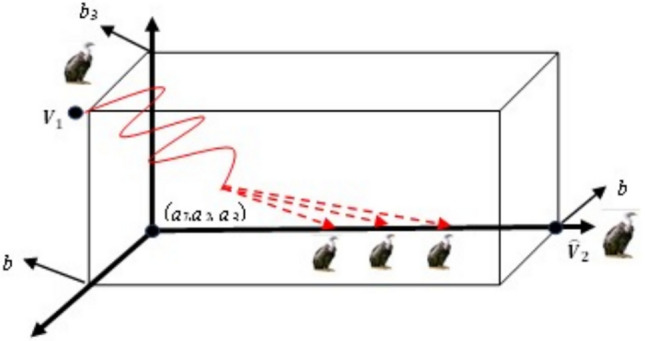
Three-dimensional EOBL mechanism space.

Mathematically, the optimization problem’s candidate solution is represented as shown in Eq. (21), in coordinate form as an m-dimensional space, and it is of the form:

21 $$\begin{array}{*{20}c} { {\hat{\text{V}}}_{{{\text{ij}}}} = \left\{ {\begin{array}{*{20}c} {{\text{N}} + \left( {{\text{rand}}^{2} {* }\frac{{{\text{V}}_{{{\text{ij}}}} }}{2}} \right),} & {{\text{norm}}\left( {{\text{V}}_{{{\text{ij}}}} } \right) \le {\text{norm}}\left( {\text{N}} \right)} \\ {{\text{N}} - \left( {{\text{rand}}^{2} {* }\frac{{{\text{V}}_{{{\text{ij}}}} }}{2}} \right),} & {{\text{norm}}\left( {{\text{V}}_{{{\text{ij}}}} } \right) > {\text{norm}}\left( {\text{N}} \right)} \\ \end{array} } \right. } \\ \end{array}$$

where $N=\frac{{\varvec{a}}+{\varvec{b}}}{2}$, ***a*** denotes the lower limit and ***b*** denotes the upper limit of the search interval, and ${rand}^{2}$ represents a small arbitrary number between [0, 1], which aids in making use of the search space’s promising areas. Additionally, this technique predetermines when to use EOBL in this algorithm by defining a jumping probability or jumping parameter (*J*_*r*_). Linking to Eq. (15), the new functions are proposed so that the existing rules are altered to avoid poor diversity and to promote convergence while avoiding local optima. Figures 10, 11, and 12, respectively, depict V and $\widehat{\text{V}}$, in all three-dimensional spaces.

### Integrates EOBL with AVO

The basis of the suggested EOBAVO technique, which seeks to increase the AVO technique’s efficiency, is described in this segment. The AVO technique is improved by merging it with the EOBL technique, which improves its ability to swiftly find the ideal value while thoroughly exploring the search space. The AVO approach has several drawbacks, such as the addition of exploitation capability to the exploration phase to speed up convergence and the random strategy that determines the conversion from the exploration phase through to the exploitation phase, which also impacts balance. This idea causes local optima trapping and affects the region’s global search, which is enhanced by EOBAVO. The suggested approach takes the opposite values when exploring the full search area by taking into account the two potential places for the calculated value to avoid these situations. This alteration raises the likelihood that the best answers will be discovered faster and more effectively. Every mathematical change must be tested, though, as the NFL theorem states that no optimization technique can successfully handle every issue^17^. There are two stages to the integration of the EOBL with the AVO. The population is initialized using EOBL in the first phase, and new vultures are developed using the data at hand in the second phase. These phases are explained in depth in the following subsections.

#### Initialize population by EOBL

In the first phase, we set the population $V_{i} = \left\{ {v_{i1} , v_{i2} , \ldots ,v_{ij} , \ldots v_{iD} } \right\}\quad \left( {i = 1, 2, 3 \ldots MP;j = 1,2,3 \ldots D} \right)$ randomly in the search space, and MP represents the population size in dimension D. The EOBL technique is used to determine the optimal value for each solution as well as the value associated with every solution in the population. The original population ${V}_{i}$ and its corresponding population ${\widehat{V}}_{i}$ will then be combined to form a single group. From $\left\{{V}_{i} , {\widehat{V}}_{i}\right\}$, we select the optimal MP result. The original population will now have the best MP solutions.

#### Update the new group of vultures

At the second phase, each revised AVO solution is restructured using the function before the modification in agreement with Eqs. (1–16). Then the fitness value and optimal solution are sustained. The EOBL is applied to create new vultures with a particular rate of probability (*J*_*r*_). An arbitrary number from 0 to 1 is then generated. Our proposed technique’s capacity for exploration is improved by this jumping parameter (*J*_*r*_). If the random value is less than J_r_, we apply EOBL to create new Vultures based on the existing population. Next, we combine the present vulture with the appropriate vulture to select the MP fittest vulture. The approach may bring to equilibrium the exploitation and exploration capacities with the probability of *J*_*r*_ = 0.1, since the EOBL can be thought of as the mutation operator.

### Time and space complexity

The computational complexity of any algorithm is a crucial component in assessing its performance. The time and space complexity of the proposed method is as follows:

i. The vulture’s population is initialized in O (n _*_ D) time, where n is the population size and D is the variable dimension. To calculate each vulture’s fit value, O(n) is needed.
ii. The formula O Max_iter _*_ n) time is used to estimate each vulture’s fitness value, where Max_iter is the maximum number of iterations.

Table 2 shows the average runtime per run over 30 independent runs for a representative set of benchmark functions (F3, F8 from CEC2005 and CEC09 from CEC2022) with a maximum of 15,000 function evaluations.

**Table 2:** Average runtime per run (in seconds).

Algorithm	F3 (CEC2005) (s)	F8 (CEC2005) (s)	CEC09 (CEC2022) (s)
AVO	1.49	3.91	5.25
OBAVO	1.65	4.17	5.49
ROBAVO	1.71	4.31	5.63
EOBAVO	1.81	4.54	5.94

From the results, EOBAVO uses approximately 8–12% extra runtime compared to standard AVO and about 5–8% compared to OBAVO/ROBAVO. This overhead stems from the extra evaluation of opposition-based solutions during initialization and occasional EOBL-based updating during the search iterations. However, the slight increase in computational cost is justified by the significant performance improvements in solution quality and convergence speed. EOBAVO consequently maintains a reasonable trade-off between computational efficiency and optimization effectiveness.

**Fig. 13:**
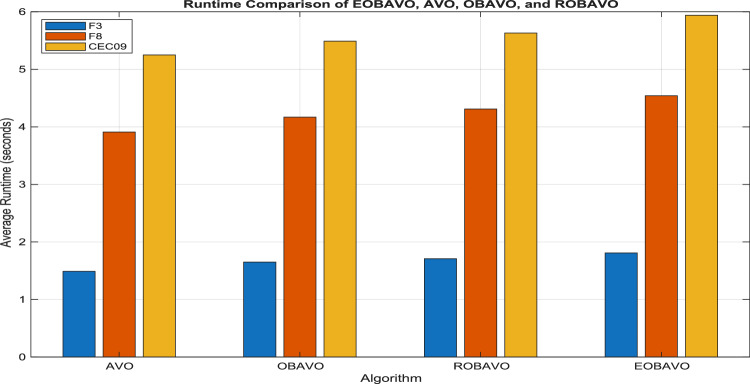
Average runtime per run.

The overall time complexity is given as: O((Max_iter_*_ n) _*_ D). From the iterations, the offspring generation takes up the most space. Therefore, O(n _*_ D) is the space complexity of EOBAVO. Figure 13 shows the graphical representation if the Average runtime per run for AVO, OBAVO, ROBAVO and EOBAVO.

### The proposed EOBAVO technique’s pseudo-code

The proposed EOBAVOA pseudo-code is given in Algorithm 1 below. We set the vulture population, ${V}_{0}$ in the search space to a random initial value.

**Algorithm 1:**
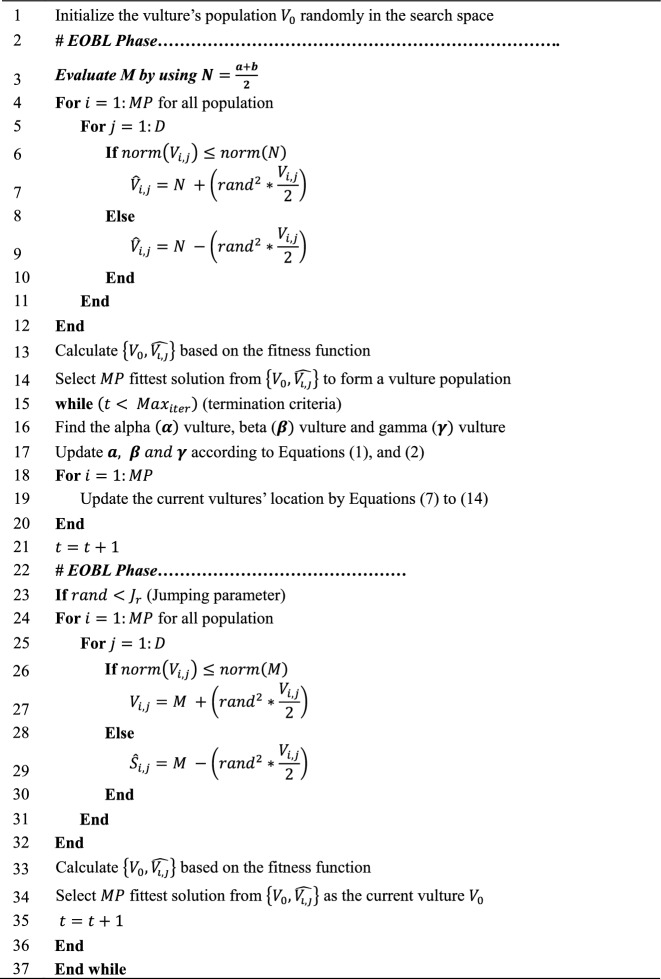
Pseudo-code of the proposed EOBAVO.

## Numerical experiments and results analysis

The proposed EOBAVO method is evaluated against AVO^16^, other Metaheuristics like AVO, PSO^4^, GSA^28^, SSA^12^, TSA^61^, MVO^35^, WVO^62^ as well as with the newest algorithms like MGO^30^, HGS^29^, and HHO^63^ and the top-performing algorithms like INFO^64^, Furthermore, the EOBAVO technique application to real-life engineering problems demonstrates how traditional AVO has improved.

### Parameter/benchmark functions settings

Assessing the effectiveness of the proposed method, 23 IEEE CEC2005 test functions^65^ and which comprise 10 fixed-dimensional multi-modal functions, six multi-modal, and seven uni-modal, have been chosen. The test functions for IEEE CEC2005 are denoted by ‘‘F’’ and their respective numbers: F1, F2, F3,…,F23. To assess an algorithm’s potential for exploitation, the unimodal test functions F1–F7 have a single global optimum solution, but F8–F13 (the multi-modal) test functions have several local optimum values. These are thought to assess a potential algorithm for exploration. The remaining fixed-dimensional multi-modal functions, F14–F23, are thought to concurrently investigate the exploration and exploitation of Metaheuristic algorithms in global and local searches because they have fewer dimensions and more local extremums than the multi-modal functions. In addition, 12 hard test functions of the IEEE CEC2022^66^ benchmark functions are applied to determine the usefulness and capabilities of the suggested technique. These functions have varying and expandable dimensions. IEEE CEC2022 test functions are denoted by ‘‘CEC,’’ which is followed by their respective numbers: CEC01, CEC02, up to CEC12. While the ranges of all the other functions are [− 100, 100], the ranges of the functions from CEC01 to CEC03 differ. The IEEE CEC2022^67^ benchmark functions are far more complicated than the IEEE CEC2005^65^ test functions that were employed in this investigation. The AVO^68^ and other prominent MAs, including SCA^33^, PSO^4^, SSA^12^, WOA^9^, were compared with the optimization outcomes of the EOBAVO. The proposed technique is also tested on a variety of real-world engineering design optimization problems. Additionally, statistical tests are used to quantify the algorithms’ statistical significance, such as the Wilcoxon rank-sum test^69^ and the t-test^70^.

Thirty search agents were used by each algorithm to look up for the search space. The average outcomes are then used for comparison. Each function is said to be performed 30 times, constrained to a maximum of 500 iterations and a total of 15,000 function evaluations (FEs). The experiment was carried out using (MATLAB R2025a, Windows 10, Intel Core i7-1165G7 CPU @ 2.80, 8GB RAM). Parameter configurations for the aforementioned algorithms are listed in Table 3 below:

**Table 3:** Mas’ parameter settings.

No	Algorithm	Parameters
1	AVO	Jumping probability (*J*_*r*_) = 0.1; a = Decrease 2 to 0 (Linearly—Convergence constant)
2	WVO	a = Decrease from 2 to 0 (Linearly)
3	SSA	Leader position updates probability = 0.5; c_1_ = Decreases linearly from 2 to 0
4	WOA	A = Decrease linearly 2:0 (Convergence constant); a_2_—Decrease linearly − 1 to − 2; and b = 1 (Spiral factor)
5	SCA	a = 2
6	GSA	Jumping probability (*J*_*r*_) = 0.1 a = Decrease linearly (2 to 0) Convergence constant
7	TSA	Jumping probability (*J*_*r*_) = 0.1 a = Decrease linearly (2 to 0) Convergence constant
8	PSO	c_1_ = 1.5 (Coefficient of Personal learning); c_2_ = 2.0 (Coefficient of Global learning); w = 1 (Inertia weights); w_damp_ = 0.99 (Ratio of Inertia damping)
9	LSHADE	Memory size = 5 (Size of memory); pbest rate = 0.11; arc rate = 1.4 (Rate of Archive)
10	DE	*pCR* = 0.2 (Crossover probability); *beta_min* = 0.2; beta_max = 0.8
11	INFO	c = 2; d = 4
12	HGS	*VC2* = 0.3 (Variable of variation control)
13	HHO	β = 1.5; *EO* = varies from − 1 to 1
14	MGO	Free parameter
15	EOBAVO	Jumping probability (*J*_*r*_) = 0.1; a = Decrease 2 to 0 (Linearly—Convergence constant)

### Performance metrics

i. Average (Avg)
  The average is the mean of an algorithm’s best results over several runs and can be determined as shown in Eq. (22):
  22 $$\begin{array}{*{20}c} {avg = \frac{1}{R}\mathop \sum \limits_{i = 1}^{R} Best_{i} } \\ \end{array}$$
  where $Best_{i}$ denotes the best result reached from the *i*th run, and the number of independent runs is denoted by R.
ii. Standard deviation (std)
  An algorithm’s repeatability and capacity to yield the same ideal outcome after multiple runs are evaluated using the standard deviation given in Eq. (23):
  23 $$\begin{array}{*{20}c} {std = \sqrt {\frac{1}{R - 1}\mathop \sum \limits_{i = 1}^{R} \left( {Best_{i} - avg} \right)^{2} } } \\ \end{array}$$
iii. The t-test
  Equation (24) shows the t-test used to assess whether a proposed method differs significantly from existing MA.
  24 $$\begin{array}{*{20}c} {t = \frac{{avg_{1} - avg_{2} }}{{\sqrt {\frac{{std_{1}^{2} - std_{2}^{2} }}{R}} }}} \\ \end{array}$$
  where ${avg}_{1}$ and ${avg}_{2}$ are the averages and ${st{d}_{1}}^{2}$ and ${st{d}_{2}}^{2}$ are also the standard deviations for any two algorithms.

### Comparison of EOBAVO with AVO, OBAVO and ROBAVO

To identify the top solution for the IEEE CEC2005^65^ and IEEE CEC2022^67^ benchmark functions, the performance of the suggested technique, EOBAVO, and the traditional AVO, OBAVO, and ROBAVO algorithms are evaluated in this section. Tables 4 and 5 present the comparison results, respectively, which show clearly that for most functions, the recommended EOBAVO approach performs better than AVO, OBAVO, and ROBAVO.

**Table 4:** The IEEE CEC2005 benchmark function results for AVO, OBAVO, ROBAVO, and EOBAVO (the top results have been highlighted).

Funs	EOBAVO		AVO		OBAVO		ROBAVO	
	Avg	Std	Avg	Std	Avg	Std	Avg	Std
F1	**0.00E+00**	**0.00E+00**	1.70E−299	0.00E**+**00	1.40E−298	0.00E**+**00	1.06E−03	5.65E−04
F2	**1.85E**−**256**	**0.00E+00**	5.90E−146	3.20E−145	9.10E−113	5.90E−128	9.00E−256	0.00E**+**00
F3	**0.00E+00**	**0.00E+00**	1.40E−214	0.00E**+**00	2.20E−210	0.00E+00	0.00E**+**00	0.00E**+**00
F4	**7.40E**−**263**	**0.00E+00**	1.00E−143	5.30E−143	1.01E−03	2.61E−04	1.60E−261	0.00E**+**00
F5	3.75E−05	**2.83E**−**05**	6.39E−05	5.19E−05	− 1.53E+04	2.90E**+**03	5.83E−05	3.83E−05
F6	3.54E−07	3.27E−07	4.22E−07	2.80E−07	0.00E**+**00	0.00E**+**00	9.19E−07	4.87E−07
F7	1.16E−04	9.45E−05	1.60E−04	1.89E−04	2.70E−14	1.30E−14	1.78E−04	1.04E−04
F8	− 1.24E**+**04	3.44E**+**02	− 1.22E**+**04	8.67E**+**02	0.00E**+**00	0.00E**+**00	− 1.56E**+**04	3.48E**+**03
F9	**0.00E+00**	**0.00E+00**	0.00E**+**00	0.00E**+**00	6.16E−04	3.40E−04	0.00E**+**00	0.00E**+**00
F10	**4.43E**−**16**	**3.01E**−**31**	4.43E−16	3.01E−31	1.41E−03	7.49E−04	4.68E−14	1.19E−15
F11	**0.00E+00**	**0.00E+00**	0.00E**+**00	0.00E**+**00	4.13E**+**00	1.22E**+**00	0.00E**+**00	0.00E**+**00
F12	**1.94E**−**08**	**1.36E**−**08**	6.09E−08	2.07E−07	1.08E−03	1.94E−04	3.22E−08	1.67E−08
F13	3.89E−08	**3.30E**−**08**	4.48E−08	5.86E−08	− 4.91E−02	9.35E−03	1.09E−07	4.68E−08
F14	1.13E**+**00	5.03E−01	1.33E**+**00	7.06E−01	7.92E−01	1.50E−01	2.75E**+**00	1.12E**+**00
F15	**3.33E**−**04**	**8.33E**−**05**	3.97E−04	1.18E−04	4.62E**+**00	8.15E−01	9.06E−04	2.67E−04
F16	− 1.03E**+**00	**0.00E+00**	−1.03E**+**00	0.00E**+**00	− 2.81E**+**00	5.33E−01	− 1.29E−01	2.50E−02
F17	3.98E−01	**1.13E**−**16**	3.98E−01	1.13E−16	− 1.95E**+**00	3.53E−01	5.70E−01	1.18E−01
F18	3.00E**+**00	**3.06E**−**06**	3.00E**+**00	3.24E−06	− 4.33E**+**00	1.25E**+**00	4.66E**+**00	9.20E−01
F19	− 3.86E**+**00	**2.53E**−**12**	− 3.86E**+**00	1.48E−11	− 1.13E**+**01	2.31E**+**00	− 2.81E**+**00	5.40E−01
F20	− 3.30E**+**00	**4.51E**−**02**	− 3.26E**+**00	6.05E−02	− 4.00E**+**00	7.21E−01	− 1.98E**+**00	3.03E−01
F21	− **1.02E+01**	5.29E−13	− 1.02E**+**01	5.15E−13	− 3.81E**+**00	7.50E−01	− 6.81E**+**00	1.57E**+**00
F22	− **1.04E+01**	3.20E−12	− 1.04E**+**01	3.32E−13	− 3.81E**+**00	7.50E−01	− 6.81E**+**00	1.57E**+**00
F23	− **1.05E+01**	**4.56E**−**13**	− 1.05E**+**01	6.41E−13	− 3.86E**+**00	7.61E−01	− 6.59E**+**00	1.35E**+**00

**Table 5:** The IEEE CEC2022 benchmark function results for AVO, OBAVO, ROBBWOA, and EOBAVO (the top results have been highlighted).

Functions	EOBAVO		AVO		OBAVO		ROBAVO	
	Avg	Std	Avg	Std	Avg	Std	Avg	Std
CEC01	6.47E**+**02	7.11E**+**02	4.88E**+**02	2.63E**+**02	2.16E**+**03	1.04E**+**03	2.20E**+**03	8.29E**+**02
CEC02	4.26E**+**02	3.27E**+**01	4.16E**+**02	2.26E**+**01	6.41E**+**02	2.74E**+**01	6.22E**+**02	2.47E**+**01
CEC03	6.23E**+**02	**1.13E+01**	6.21E**+**02	1.13E**+**01	7.67E**+**02	4.07E**+**01	7.63E**+**02	3.69E+01
CEC04	**8.29E+02**	**5.94E+00**	8.34E+02	1.13E+01	9.80E+02	3.73E+01	9.73E+02	3.70E+01
CEC05	**1.33E+03**	1.38E+02	1.34E+03	2.03E+02	1.58E+03	1.43E+02	1.61E+03	1.26E+02
CEC06	**3.19E+03**	**1.44E+03**	3.52E+03	1.72E+03	4.83E+03	1.50E+03	4.48E+03	1.59E+03
CEC07	**2.05E+03**	**2.44E+01**	2.05E+03	2.75E+01	2.26E+03	2.80E+01	2.16E+03	2.69E+01
CEC08	**2.23E+03**	**9.65E+00**	2.23E+03	9.57E+00	2.54E+03	1.31E+01	2.34E+03	1.23E+01
CEC09	2.55E+03	3.90E+01	2.54E+03	1.31E+01	2.90E+03	4.74E+01	2.72E+03	3.64E+01
CEC10	2.57E+03	6.66E+01	2.55E+03	6.79E+01	2.86E+03	4.17E+01	2.76E+03	4.09E+01
CEC11	**2.71E+03**	**1.46E+02**	2.74E+03	1.61E+02	2.96E+03	1.75E+02	2.98E+03	1.79E+02
CEC12	**2.87E+03**	1.03E+01	2.87E+03	8.65E+00	3.08E+03	1.20E+01	3.27E+03	1.66E+02

Table 4 shows that the EOBAVO produced a better result than the rest, i.e., AVO, OBAVO, and ROBAVO, for functions F1–F4, F9–F15, F17–F19, and F21–F23. Functions F1, F3, F9, and F11 show successful exact global optimal solutions. The standard deviation measure for the proposed EOBAVO technique is higher than that of the classical AVO, OBAVO, and ROBAVO.

The average and standard deviation in Table 5 indicate that the EOBAVO technique performs better than the other three algorithms, except for the functions CEC01- CEC03, CEC09, and CEC10.

### Evaluation of EOBAVO with other innovative and successful algorithms

A comparison of the effectiveness of the EOBAVO technique with four traditional, well-known Metaheuristic techniques: SCA, PSO, SSA, and WOA, was done in this section. Table 1 lists these algorithms’ parameter configurations. Table 6 shows that for the average fitness values, the EOBAVO approach fared better than any other algorithm of the IEEE CEC2005^65^.

**Table 6:** IEEE CEC2005 benchmark function results for SCA, PSO, SSA, WOA, and EOBAVO (the top results are in Bold).

Fun	EOBAVO		SCA		PSO		SSA		WOA	
	Avg	Std	Avg	Std	Avg	Std	Avg	Std	Avg	Std
F1	**0.00E+00**	**0.00E+00**	9.25E−12	3.29E−11	1.90E−107	1.10E−106	3.96E−07	8.64E−07	2.53E−16	9.67E−17
F2	**1.85E−256**	**0.00E+00**	4.42E−09	1.54E−08	2.93E−57	1.26E−56	9.05E−02	4.51E−01	5.57E−02	1.94E−01
F3	**0.00E+00**	**0.00E+00**	4.50E−02	1.68E−01	7.24E−78	3.89E−77	1.76E−06	5.49E−06	8.97E+02	3.19E+02
F4	**7.40E−263**	**0.00E+00**	1.22E−03	2.59E−03	8.51E−49	2.19E−48	6.36E−05	2.17E−04	7.35E+00	1.74E+00
F5	3.75E−05	**2.83E−05**	7.95E+00	2.55E+00	2.77E+01	7.62E−01	9.47E+01	1.45E+02	6.75E+01	6.22E+01
F6	3.54E−07	3.27E−07	4.94E−01	1.53E−01	2.77E−01	1.83E−01	1.20E−09	4.41E−10	2.50E−16	1.74E−16
F7	1.16E−04	9.45E−05	3.54E−03	2.82E−03	8.50E−04	8.02E−04	1.35E−02	6.78E−03	8.94E−02	4.34E−02
F8	− 1.24E+04	3.44E+02	− 2.15E+03	1.65E+02	− 6.81E+03	9.72E+02	− 2.67E+03	3.71E+02	−2.81E+03	3.93E+02
F9	**0.00E+00**	**0.00E+00**	7.47E−01	2.80E+00	0.00E+00	0.00E+00	1.59E+01	6.70E+00	2.60E+01	7.47E+00
F10	**4.44E−16**	**3.01E−31**	7.15E−07	2.18E−06	8.88E−16	1.00E−31	9.59E−01	8.74E−01	6.21E−02	2.36E−01
F11	**0.00E+00**	**0.00E+00**	6.71E−02	1.24E−01	0.00E+00	0.00E+00	1.99E−01	1.28E−01	2.77E+01	5.04E+00
F12	**1.94E−08**	**1.36E−08**	1.00E−01	2.69E−02	1.28E−02	2.09E−02	6.53E−01	9.09E−01	1.80E+00	9.51E−01
F13	**3.89E−08**	**3.30E−08**	3.30E−01	8.80E−02	1.26E+00	5.72E−01	5.17E−02	5.80E−03	8.90E+00	7.13E+00
F14	**1.13E+00**	5.03E−01	1.60E+00	9.23E−01	1.91E+00	2.01E+00	1.22E+00	6.73E−01	5.86E+00	3.83E+00
F15	**3.33E−04**	**8.33E−05**	1.04E−03	3.65E−04	1.94E−03	5.33E−03	5.17E−03	9.32E−03	3.67E−03	1.65E−03
F16	− 1.04E+00	**0.00E+00**	− 1.02E+00	4.87E−05	−1.02E+00	1.07E−08	− 1.03E+00	2.76E−14	− 1.03E+00	4.88E−16
F17	**3.97E−01**	**1.12E−16**	4.00E−01	2.35E−03	3.97E−01	1.13E−16	3.97E−01	1.35E−14	3.97E−01	0.00E+00
F18	**3.01E+00**	**3.06E−06**	3.01E+00	4.70E−05	3.01E+00	8.35E−15	3.01E+00	1.91E−13	3.01E+00	4.17E−15
F19	− **3.87E+00**	**2.53E−12**	− 3.85E+00	2.29E−03	− 3.86E+00	4.05E−15	− 3.86E+00	4.07E−10	− 3.86E+00	2.29E−15
F20	− 3.30E+00	**4.51E−02**	− 2.85E+00	3.73E−01	− 3.26E+00	6.05E−02	− 3.21E+00	5.68E−02	− 3.32E+00	2.31E−02
F21	− **1.01E+01**	5.29E−13	− 2.20E+00	1.86E+00	− 8.05E+00	2.89E+00	− 7.73E+00	3.12E+00	− 5.96E+00	3.74E+00
F22	− **1.04E+01**	3.20E−12	− 3.18E+00	1.82E+00	− 9.60E+00	2.09E+00	− 7.56E+00	3.39E+00	− 9.68E+00	2.01E+00
F23	− **1.05E+01**	**4.56E−13**	− 3.33E+00	1.73E+00	− 9.54E+00	2.58E+00	− 7.60E+00	3.51E+00	− 1.05E+01	2.60E−15

Nonetheless, the EOBAVO approach produced better and absolute global optimal solutions for the functions F16, F19, and F20. Except for F6, F8, F13, F14, and F16–F19, the suggested algorithm fared better than most functions in terms of the standard deviation. Table 7 shows that, compared to alternative methods for the IEEE CEC2022^81^ test functions, the suggested EOBAVO methodology is superior at solving them. For example, Table 5 shows that the suggested technique outperformed the others for the functions F1, F3, F5, F8, and F10 in terms of average fitness value. Additionally, when the standard deviations of other methods are compared, EOBAVO functions F1–F2, F5, and F7–F8 have shown better outcomes.

**Table 7:** The IEEE CEC2022 benchmark function results for AVO, OBAVO, ROBBWOA, and EOBAVO (the top results have been highlighted).

Func.	EOBAVO		SCA		PSO		SSA		WOA	
	Avg	Std	Avg	Std	Avg	Std	Avg	Std	Avg	Std
CEC01	6.47E+02	7.11E+02	1.33E+12	9.98E+11	2.28E+12	1.18E+04	− 7.26E+05	1.79E+06	9.64E+05	4.86E+03
CEC02	4.26E+02	3.27E+01	1.29E+04	3.57E+03	2.39E+12	2.20E+04	− 3.00E+05	3.38E+05	− 2.12E+05	5.31E+05
CEC03	6.23E+02	**1.13E+01**	1.38E+01	9.13E−15	1.75E+05	1.86E+04	5.82E+05	6.82E+05	1.41E+06	1.83E+05
CEC04	**8.29E+02**	**5.94E+00**	1.84E+01	7.85E+00	4.33E+11	1.60E+04	5.45E+05	8.62E+03	2.64E+06	4.97E+05
CEC05	**1.33E+03**	1.38E+02	1.14E+11	1.25E−01	9.01E+11	1.23E+04	1.23E+06	2.58E+05	4.39E+05	3.13E+05
CEC06	**3.18E+03**	**1.44E+03**	9.20E+00	1.76E+00	1.34E+11	9.82E+03	3.43E+05	− 3.20E+04	− 4.47E+05	1.72E+06
CEC07	**2.05E+03**	**2.44E+01**	2.12E+02	1.57E+02	2.33E+08	1.44E+04	1.99E+05	− 5.83E+05	− 2.03E+05	− 1.43E+05
CEC08	**2.23E+03**	**9.65E+00**	5.47E+00	4.73E−01	1.59E+12	1.21E+04	− 1.14E+06	6.17E+05	7.19E+05	− 3.78E+05
CEC09	2.55E+03	3.90E+01	2.47E+06	1.82E−02	2.17E+12	8.15E+03	− 4.26E+05	− 6.60E+05	− 5.01E+05	− 1.05E+06
CEC10	2.57E+03	6.66E+01	2.15E+04	1.42E−01	8.34E+11	1.29E+04	− 4.68E+05	− 1.78E+06	− 7.89E+05	2.63E+05
CEC11	**2.71E+03**	**1.46E+02**	3.13E+03	1.26E+00	3.24E+12	9.74E+03	− 7.87E+05	1.49E+05	1.41E+05	− 1.04E+06
CEC12	**2.87E+03**	1.03E+01	1.48E+08	8.21E+04	3.22E+07	1.70E+04	2.69E+06	− 2.13E+06	1.81E+06	− 5.60E+04

### Analysis of sensitivity

The Sensitivity analysis for MAs looks at how several independent variables’ values impact a specific outcome. This is also a crucial part of parameter tuning. The proposed EOBAVO technique is used to analyze the sensitivity of *J*_*r*_ (the jumping parameter) and *rand*^*2*^. Below is a discussion of five different scenarios.

#### Analysis of J_r_

**Fig. 14:**
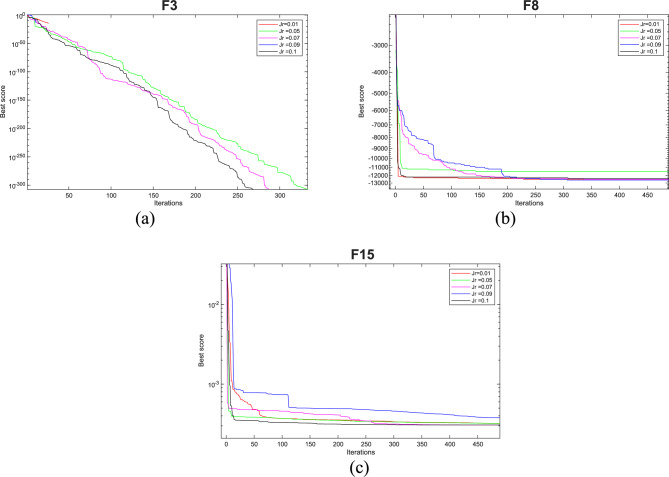
Sensitivity analysis of the EOBAVO algorithm for jumping parameter.

The EOBAVO technique is used with different *J*_*r*_ values, while we keep other parameters constant, to analyze the importance of the parameter. *J*_*r*_ will take the following values: 0.01; 0.05; 0.07; 0.09; and 0.1. The reasons for these close values are that small, closely spaced values permit detailed performance assessment to guarantee fine-grained sensitivity analysis. It also prevents undue disturbances while harmonizing exploration and exploitation (controlled randomness). The functions F3, F8, and F15 are a subgroup of test functions selected from each category. With a maximum of 15,000 FEs, each function executes 30 times. Table 8 and Fig. 14 show the outcomes that were recorded for each case. Results show that when *J*_*r*_ is 0.1, the EOBAVO approach gave the best results.

**Table 8:** Sensitivity study of EOBAVO with varying parameter Jr. values

Parameter scenarios *J*_*r*_	F3	F8	F15
0.01	1.8206 E−275	**− **1.24E+04	3.21E−04
0.05	0	**− **1.14E+04	3.20E−04
0.07	0	**− **1.25E+04	3.08E−04
0.09	0	**− **1.26E+04	3.77E−04
0.1	0	− 1.26E+04	3.08E−04

#### Analysis of rand ^2^

**Fig. 15:**
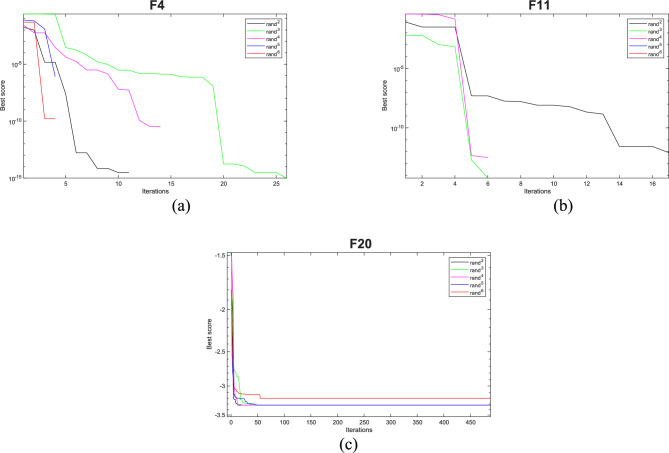
Sensitivity analysis of the proposed EOBAVO algorithm for rand^2^.

The EOBAVO technique is used with several different scenarios, such as *rand*^*3*^, *rand*^*4*^, *rand*^*5*^, and *rand*^*6*^, and keeping all factors constant to analyze the relevance of the *rand*^*2*^ parameter. F4, F11, and F20 are a subset of test functions selected from each category. With a maximum of 15,000 FEs, each function executes 30 times. The statistical results for each state are shown in Table 9 and Fig. 15. It is clear from the experimental results that *rand*^*2*^ solves issues better than the others.

**Table 9:** Sensitivity study of EOBAVO with varying parameter *rand*^*2*^ values.

Parameter scenarios	F4	F11	F20
*rand*^*2*^	6.55E−304	**0**	**− 3.32E + 00**
*rand*^*3*^	0	0	**− **3.32E + 00
*rand*^*4*^	0	0	**− **3.32E + 00
*rand*^*5*^	0	0	**− **3.32E + 00
*rand*^*6*^	0	0	**− **3.20E + 00

### Statistical analysis

Here, the effectiveness of the suggested EOBAVO technique is estimated using the Wilcoxon rank-sum test^71^ and the t-test^69^. In calculating the t-values for the function in a t-test, the two algorithms are considered simultaneously. Tables 10 and 11 present the t-test results at α = 0.05% for test functions (12 test functions) from IEEE CEC2022^66^ and 23 test functions from IEEE CEC2005^65^. The other methods are outperformed by EOBAVO if the relevant t-value is bold-faced. Additionally, the last row of Tables 10 and 11 shows EOBAVO’s win, tie, and loss counts, which are labelled as w = t = l. The t-values make it clear that, in many situations, EOBAVO performance is greatly enhanced.

**Table 10:** Findings from the IEEE CEC2005 benchmark functions for unimodal, multimodal, and fixed-dimensional multimodal (the top solutions have been highlighted).

Func		SCA	PSO	SSA	WOA	AVO	OBAVO	ROBAVO	EOBAVO
F1	Mean	3.84E−08	1.90E−107	3.96E−07	2.53E−16	1.70E−299	1.40E−298	1.60E−298	0.00E+00
	Std	7.94E−08	1.10E−106	8.64E−07	9.67E−17	0.00E+00	0.00E+00	0.00E+00	0.00E+00
	t-values	**− 2.65E+00**	**− 1.00E+00**	**− 2.51E+00**	**− 1.43E+01**	**#DIV/0!**	**#DIV/0!**	**#DIV/0!**	NA
F2	Mean	5.04E−02	2.93E−57	9.05E−02	5.57E−02	5.90E−146	9.10E−113	1.00E−112	1.85E−256
	Std	6.23E−02	1.26E−56	4.51E−01	1.94E−01	3.20E−145	5.90E−128	5.80E−128	0.00E+00
	t-values	**− 4.43E+00**	**− 1.28E+00**	**− 1.10E+00**	**− 1.57E+00**	**− 1.00E+00**	**− 8.40E+15**	**− 9.50E+15**	NA
F3	Mean	8.17E+01	7.24E−78	1.76E−06	8.97E+02	1.40E−214	2.20E−210	2.40E−210	0.00E+00
	Std	5.87E+01	3.89E−77	5.49E−06	3.19E+02	0.00E+00	0.00E+00	0.00E+00	0.00E+00
	t-values	**− 7.63E+00**	**− 1.02E+00**	**− 1.76E+00**	**− 1.54E+01**	**#DIV/0!**	**#DIV/0!**	**#DIV/0!**	NA
F4	Mean	2.15E+00	8.51E−49	6.36E−05	7.35E+00	1.00E−143	1.01E−03	3.01E−03	7.40E−263
	Std	8.27E−01	2.19E−48	2.17E−04	1.74E+00	5.30E−143	2.61E−04	3.61E−04	0.00E+00
	t-values	**− 1.42E+01**	**− 2.13E+00**	**− 1.60E+00**	**− 2.31E+01**	**− 1.05E+00**	**− 2.12E+01**	**− 4.57E+01**	NA
F5	Mean	4.97E+01	2.77E+01	9.47E+01	6.75E+01	6.39E−05	−1.53E+04	−1.63E+04	3.75E−05
	Std	3.76E+01	7.62E−01	1.45E+02	6.22E+01	5.19E−05	2.90E+03	1.90E+03	2.83E−05
	t-values	**− 7.23E+00**	**− 1.99E+02**	**− 3.58E+00**	**− 5.95E+00**	**− 2.45E+00**	**2.89E+01**	**4.70E+01**	NA
F6	Mean	2.46E−05	2.77E−01	1.19E−09	2.50E−16	4.22E−07	0.00E+00	0.00E+00	3.54E−07
	Std	1.19E−04	1.84E−01	4.41E−10	1.74E−16	2.80E−07	0.00E+00	0.00E+00	3.27E−07
	t-values	**− 1.11E+00**	**− 8.22E+00**	**5.91E+00**	**5.93E+00**	**− 8.69E−01**	**5.93E+00**	**5.93E+00**	NA
F7	Mean	3.01E−02	8.50E−04	1.35E−02	8.94E−02	1.60E−04	2.70E−14	1.35E+05	1.15E−04
	Std	1.85E−02	8.02E−04	6.78E−03	4.34E−02	1.89E−04	1.30E−14	1.49E+03	9.45E−05
	t-values	**− 8.88E+00**	**− 4.98E+00**	**− 1.08E+01**	**− 1.13E+01**	**− 1.14E+00**	**6.70E+00**	**− 4.98E+02**	NA
F8	Mean	−6.53E+03	−6.82E+03	−2.67E+03	−2.82E+03	−1.22E+04	0.00E+00	0.00E+00	−1.24E+04
	Std	7.43E+02	9.72E+02	3.71E+02	4.93E+02	8.67E+02	0.00E+00	0.00E+00	3.44E+02
	t-values	**− 3.92E+01**	**− 2.96E+01**	**− 1.05E+02**	**− 8.71E+01**	**− 1.19E+00**	**− 1.97E+02**	**− 1.97E+02**	NA
F9	Mean	5.02E+01	0.00E+00	1.59E+01	2.60E+01	0.00E+00	6.16E−04	5.26E−04	0.00E+00
	Std	1.32E+01	0.00E+00	6.70E+00	7.47E+00	0.00E+00	3.40E−04	4.20E−04	0.00E+00
	t-values	**− 2.09E+01**	**#DIV/0!**	**− 1.30E+01**	**− 1.90E+01**	**#DIV/0!**	**− 9.91E+00**	**− 6.85E+00**	NA
F10	Mean	1.65E+00	8.88E−16	9.59E−01	6.21E−02	4.44E−16	1.41E−03	2.51E−03	4.44E−16
	Std	7.01E−01	1.00E−31	8.74E−01	2.36E−01	3.01E−31	7.49E−04	7.89E−04	3.01E−31
	t-values	**− 1.29E+01**	**− 7.70E+15**	**− 6.01E+00**	**− 1.44E+00**	**0.00E+00**	**− 1.03E+01**	**− 1.74E+01**	NA
F11	Mean	3.72E−02	0.00E+00	1.99E−01	2.77E+01	0.00E+00	4.13E+00	1.36E+05	0.00E+00
	Std	3.61E−02	0.00E+00	1.28E−01	5.04E+00	0.00E+00	1.22E+00	1.58E+03	0.00E+00
	t-values	**− 5.64E+00**	**#DIV/0!**	**− 8.52E+00**	**− 3.01E+01**	**#DIV/0!**	**− 1.85E+01**	**− 4.73E+02**	NA
F12	Mean	7.60E−02	1.28E−02	6.53E−01	1.80E+00	6.09E−08	1.08E−03	2.17E−03	1.94E−08
	Std	1.39E−01	2.09E−02	9.09E−01	9.51E−01	2.07E−07	1.94E−04	2.34E−04	1.36E−08
	t-values	**− 3.00E+00**	**− 3.35E+00**	**− 3.94E+00**	**− 1.04E+01**	**− 1.09E+00**	**− 3.05E+01**	**− 5.08E+01**	NA
F13	Mean	2.11E−01	1.26E+00	5.17E−02	8.90E+00	4.48E−08	−4.91E−02	−5.50E+16	3.89E−08
	Std	7.18E−01	5.72E−01	5.80E−03	7.13E+00	5.86E−08	9.35E−03	5.45E−03	3.30E−08
	t-values	**− 1.61E+00**	**− 1.21E+01**	**− 4.88E+01**	**− 6.84E+00**	**− 4.81E−01**	**2.87E+01**	**5.49E+19**	NA
F14	Mean	3.69E+00	1.92E+00	1.23E+00	5.86E+00	1.33E+00	7.92E−01	9.89E−01	1.13E+00
	Std	2.62E+00	2.01E+00	6.73E−01	3.83E+00	7.06E−01	1.50E−01	3.45E−01	5.03E−01
	t-values	**− 5.25E+00**	**− 2.08E+00**	**− 6.46E−01**	**− 6.70E+00**	**− 1.25E+00**	**3.53E+00**	**1.27E+00**	NA
F15	Mean	1.10E−03	1.95E−03	5.17E−03	3.67E−03	3.97E−04	4.62E+00	5.33E+00	3.33E−04
	Std	3.65E−03	5.33E−03	9.32E−03	1.65E−03	1.18E−04	8.15E−01	9.21E−01	8.33E−05
	t-values	**− 1.15E+00**	**− 1.66E+00**	**− 2.84E+00**	**− 1.11E+01**	**− 2.40E+00**	**− 3.10E+01**	**− 3.17E+01**	NA
F16	Mean	−1.03E+00	−1.03E+00	−1.03E+00	−1.03E+00	−1.03E+00	−2.81E+00	−2.81E+00	−1.03E+00
	Std	1.00E−11	1.07E−08	2.76E−14	4.88E−16	0.00E+00	5.33E−01	5.33E−01	0.00E+00
	t-values	**0.00E+00**	**−1.49E+00**	**−4.94E+00**	**1.74E+10**	**#DIV/0!**	**1.83E+01**	**1.83E+01**	NA
F17	Mean	3.98E−01	3.98E−01	3.98E−01	3.98E−01	3.98E−01	−1.95E+00	−1.95E+00	3.98E−01
	Std	1.13E−16	1.13E−16	1.35E−14	0.00E+00	1.13E−16	3.53E−01	3.53E−01	1.13E−16
	t-values	**0.00E+00**	**0.00E+00**	**−4.12E+00**	**1.74E+10**	**0.00E+00**	**3.64E+01**	**3.64E+01**	NA
F18	Mean	3.00E+00	3.00E+00	3.00E+00	3.00E+00	3.00E+00	−4.33E+00	−4.33E+00	3.00E+00
	Std	3.03E−15	8.35E−15	1.91E−13	4.17E−15	3.24E−06	1.25E+00	1.25E+00	3.06E−06
	t-values	**3.40E+00**	**3.40E+00**	**3.40E+00**	**3.40E+00**	**−2.35E−01**	**3.21E+01**	**3.21E+01**	NA
F19	Mean	**− **3.00E−01	**− **3.86E+00	**− **3.86E+00	**− **3.86E+00	**− **3.86E+00	**− **1.13E+01	**− **1.13E+01	**− **3.86E+00
	Std	1.00E−11	4.05E−15	4.07E−10	2.29E−15	1.48E−11	2.31E+00	2.31E+00	2.53E−12
	t-values	**− 1.90E+12**	**1.30E+00**	**− 1.02E+00**	**− 4.64E+06**	**− 9.70E−01**	**1.77E+01**	**1.77E+01**	NA
F20	Mean	−3.29E+00	−3.26E+00	−3.21E+00	−3.32E+00	−3.26E+00	−4.00E+00	−4.00E+00	−3.30E+00
	Std	5.54E−02	6.05E−02	5.68E−02	2.31E−02	6.05E−02	7.21E−01	7.21E−01	4.51E−02
	t-values	**− 1.22E+00**	**− 2.88E+00**	**− 6.73E+00**	**1.69E+00**	**− 2.88E+00**	**5.30E+00**	**5.30E+00**	NA
F21	Mean	−5.49E+00	−8.05E+00	−7.73E+00	−5.96E+00	−1.02E+01	−3.81E+00	−3.81E+00	−1.02E+01
	Std	3.45E+00	2.89E+00	3.12E+00	3.74E+00	5.15E−13	7.50E−01	7.50E−01	5.29E−13
	t-values	**− 7.41E+00**	**− 3.99E+00**	**− 4.26E+00**	**− 6.15E+00**	**1.19E−01**	**− 4.63E+01**	**− 4.63E+01**	NA
F22	Mean	−7.84E+00	−9.60E+00	−7.56E+00	−9.68E+00	−1.04E+01	−3.81E+00	−3.81E+00	−1.04E+01
	Std	3.45E+00	2.09E+00	3.39E+00	2.01E+00	3.32E−13	7.50E−01	7.50E−01	3.20E−12
	t-values	**− 4.06E+00**	**− 2.10E+00**	**− 4.58E+00**	**− 1.95E+00**	**1.19E+00**	**− 4.81E+01**	**− 4.81E+01**	NA
F23	Mean	−8.14E+00	−9.54E+00	−7.60E+00	−1.05E+01	−1.05E+01	−3.86E+00	−3.86E+00	−1.05E+01
	Std	3.52E+00	2.58E+00	3.51E+00	2.60E−15	6.41E−13	7.61E−01	7.61E−01	4.56E−13
	t-values	**− 3.73E+00**	**− 2.11E+00**	**− 4.59E+00**	**− 1.20E+08**	**− 2.72E−01**	**− 4.80E+01**	**− 4.80E+01**	NA
	w/t/l	16.6.1	12.10.1	16.5.2	16.2.5	3.18.2	10.3.10	12.4.7

**Table 11:** Findings from the IEEE CEC2022 benchmark functions (the top answers are indicated).

Func		SCA	PSO	SSA	WOA	AVO	OBAVO	ROBAVO	EOBAVO
CEC01	Mean	1.328E+12	2.277E+12	− 7.257E+05	9.641E+05	4.883E+02	2.165E+03	2.198E+03	6.466E+02
	Std	9.984E+11	1.177E+04	1.792E+06	4.859E+03	2.632E+02	1.037E+03	8.290E+02	7.114E+02
	t-values	− 7.285E+00	− 1.058E+09	2.220E+00	− 1.075E+03	1.143E+00	− 6.612E+00	− 7.779E+00	NA
CEC02	Mean	1.287E+04	2.385E+12	− 3.008E+05	− 2.117E+05	4.159E+02	6.412E+02	6.225E+02	4.265E+02
	Std	3.573E+03	2.204E+04	3.383E+05	5.320E+05	2.262E+01	2.736E+01	2.473E+01	3.268E+01
	t- values	− 1.907E+01	− 5.927E+08	4.877E+00	2.184E+00	1.451E+00	− 2.760E+01	− 2.620E+01	NA
CEC03	Mean	1.380E+01	1.753E+05	5.822E+05	1.405E+06	6.212E+02	7.666E+02	7.628E+02	6.229E+02
	Std	9.125E− 15	1.860E+04	6.818E+05	1.828E+05	1.132E+01	4.073E+01	3.694E+01	1.133E+01
	t-values	2.945E+02	− 5.144E+01	− 4.672E+00	− 4.210E+01	5.955E− 01	− 1.861E+01	− 1.982E+01	NA
CEC04	Mean	1.836E+01	4.334E+11	5.452E+05	2.637E+06	8.336E+02	9.795E+02	9.735E+02	8.288E+02
	Std	7.852E+00	1.599E+04	8.622E+03	4.966E+05	1.134E+01	3.734E+01	3.695E+01	5.937E+00
	t- values	4.509E+02	− 1.485E+08	− 3.458E+02	− 2.907E+01	− 2.060E+00	− 2.183E+01	− 2.117E+01	NA
CEC05	Mean	1.146E+11	9.013E+11	1.238E+06	4.387E+05	1.337E+03	1.576E+03	1.606E+03	1.326E+03
	Std	1.260E− 01	1.238E+04	2.576E+05	3.125E+05	2.028E+02	1.428E+02	1.261E+02	1.383E+02
	t-values	− 4.538E+09	− 3.989E+08	− 2.630E+01	− 7.667E+00	− 2.501E− 01	− 6.881E+00	− 8.211E+00	NA
CEC06	Mean	9.201E+00	1.352E+11	3.429E+05	− 4.472E+05	3.518E+03	4.831E+03	4.483E+03	3.192E+03
	Std	1.764E+00	9.819E+03	− 3.198E+04	1.721E+06	1.722E+03	1.503E+03	1.593E+03	1.435E+03
	t-values	1.215E+01	− 7.461E+07	− 5.813E+01	1.433E+00	− 7.964E− 01	− 4.318E+00	− 3.296E+00	NA
CEC07	Mean	2.124E+02	2.327E+08	1.988E+05	− 2.032E+05	2.053E+03	2.259E+03	2.164E+03	2.046E+03
	Std	1.568E+02	1.442E+04	− 5.827E+05	− 1.427E+05	2.754E+01	2.799E+01	2.685E+01	2.437E+01
	t-values	6.328E+01	− 8.839E+04	− 1.849E+00	7.879E+00	− 1.039E+00	− 3.154E+01	− 1.782E+01	NA
CEC08	Mean	5.468E+00	1.594E+12	− 1.142E+06	7.188E+05	2.228E+03	2.539E+03	2.338E+03	2.228E+03
	Std	4.726E− 01	1.214E+04	6.171E+05	− 3.779E+05	9.567E+00	1.311E+01	1.230E+01	9.651E+00
	t-values	1.260E+03	− 7.192E+08	1.016E+01	− 1.039E+01	8.574E− 02	− 1.047E+02	− 3.838E+01	NA
CEC09	Mean	2.466E+06	2.166E+12	− 4.264E+05	− 5.011E+05	2.538E+03	2.904E+03	2.718E+03	2.547E+03
	Std	1.823E− 02	8.146E+03	− 6.602E+05	− 1.052E+06	1.308E+01	4.735E+01	3.638E+01	3.897E+01
	t-values	− 3.462E+05	− 1.457E+09	3.559E+00	2.622E+00	1.264E+00	− 3.189E+01	− 1.752E+01	NA
CEC10	Mean	2.145E+04	8.337E+11	− 4.681E+05	− 7.886E+05	2.551E+03	2.856E+03	2.759E+03	2.571E+03
	Std	1.423E− 01	1.288E+04	− 1.782E+06	2.634E+05	6.792E+01	4.167E+01	4.089E+01	6.661E+01
	t-values	− 1.552E+03	− 3.545E+08	1.447E+00	1.645E+01	1.102E+00	− 1.988E+01	− 1.318E+01	NA
CEC11	Mean	3.132E+03	3.244E+12	− 7.876E+05	1.397E+05	2.743E+03	2.964E+03	2.979E+03	2.713E+03
	Std	1.261E+00	9.737E+03	1.491E+05	− 1.045E+06	1.605E+02	1.755E+02	1.786E+02	1.463E+02
	t-values	− 1.567E+01	− 1.825E+09	2.902E+01	− 7.182E− 01	− 7.484E− 01	− 6.015E+00	− 6.290E+00	NA
CEC12	Mean	1.482E+08	3.217E+07	2.690E+06	1.811E+06	2.870E+03	3.082E+03	3.267E+03	2.870E+03
	Std	8.214E+04	1.702E+04	− 2.132E+06	− 5.599E+04	8.649E+00	1.199E+01	1.661E+02	1.031E+01
	t-values	− 9.882E+03	− 1.035E+04	− 6.905E+00	− 1.769E+02	1.795E− 01	− 7.337E+01	− 1.306E+01	NA
w/t/l		5.4.3	8.4.0	6.0.6	7.1.4	4.5.3	11.1.0	12.0.0	

A non- parametric Wilcoxon rank- sum test performed in pairs can be used to identify the significant differences in the behaviors of the two algorithms. Tables 12 and 13 present the *p* values at the a = 0.05% significant level, respectively. For two methods to be considered statistically significant, their *p* values must be less than 0.05. The results of *p* values and H are displayed in Tables 12 and 13, where the symbols ‘‘−’’ and ‘‘+’’ stand for rejection and acceptance, respectively.

**Table 12:** The IEEE CEC2005 test functions’ *P* values at the 5% significant level using the Wilcoxon rank- sum test.

Func	SCA vs EOBAVO		PSO vs EOBAVO		WOA vs EOBAVO		SSA vs EOBAVO		AVO vs EOBAVO		OBAVO vs EOBAVO		ROBAVO vs EOBAVO	
	*P *values	H	*P *values	H	*P *values	H	*P *values	H	*P *values	H	*P *values	H	*P *values	H
F1	1.40E−08	+	1.13E−08	+	5.79E−06	+	1.02E−06	+	6.32E−06	+	1.01E−05	+	9.33E−06	+
F2	7.85E−09	+	8.72E−09	+	1.50E−05	+	4.64E−06	+	3.36E−01	−	3.97E−06	+	9.58E−06	+
F3	6.95E−09	+	1.29E−08	+	7.94E−06	+	1.35E−05	+	5.40E−06	+	1.71E−06	+	4.69E−01	−
F4	4.58E−09	+	8.71E−09	+	1.12E−05	+	1.25E−05	+	3.54E−06	+	2.56E−01	−	1.29E−01	−
F5	9.45E−10	+	1.43E−08	+	2.89E−06	+	3.0915E 00	−	5.49E−06	+	4.12E−01	−	3.13E−01	−
F6	1.45E−08	+	9.25E−09	+	3.22E−06	+	3.51E−06	+	2.01E−06	+	2.69E−01	+	5.42E−06	+
F7	1.36E−08	+	9.40E−09	+	6.85E−07	+	6.21E−06	+	1.49E−05	+	3.13E−06	+	7.13E−06	+
F8	1.67E−08	+	1.47E−08	+	2.83E−06	+	8.12E−06	+	9.95E−06	+	4.78E−06	+	8.50E−06	+
F9	1.67E−08	+	6.76E−09	+	4.67E−06	+	1.04E−05	+	1.14E−05	+	2.90E−06	+	4.06E−01	−
10	9.31E−09	+	2.25E−09	+	2.97E−06	+	6.18E−06	+	1.32E−05	+	1.50E−05	+	1.92E−01	−
F11	1.29E−08	+	4.82E−10	+	1.49E−06	+	7.75E−06	+	8.35E−06	+	1.34E−06	+	1.02E−05	+
F12	1.58E−08	+	1.27E−08	+	2.02E−06	+	1.25E−05	+	1.46E−06	+	8.79E−06	+	4.84E−06	+
F13	1.42E−08	+	1.04E−08	+	NA	−	6.15E−07	+	NA	−	6.88E−06	+	9.74E−06	+
F14	4.14E−01	−	1.18E−08	+	3.46E−06	+	4.23E−06	+	9.83E−01	−	1.65E−05	+	2.59E−06	+
F15	7.55E−09	+	3.57E−09	+	6.10E−06	+	1.20E−05	+	1.25E−05	+	1.88E−06	+	8.06E−06	+
F16	2.16E−09	+	2.28E−09	+	8.43E−06	+	1.50E−05	+	7.23E−06	+	6.67E−06	+	8.92E−06	+
F17	1.60E−08	+	2.44E−10	+	1.16E−01	+	8.57E−06	+	2.14E−06	+	1.62E−05	+	8.68E−07	+
F18	1.02E−08	+	5.87E−09	+	6.59E−07	+	8.92E−01	−	4.75E−06	+	1.45E−05	+	NA	−
F19	3.83E−09	+	9.88E−09	+	1.34E−05	+	1.80E−01	−	6.08E−06	+	1.37E−01	−	NA	−
F20	1.13E−08	+	6.57E−09	+	1.05E−05	+	7.50E−06	+	1.08E−05	+	4.32E−06	+	1.06E−06	+
F21	NA	−	7.33E−09	+	1.37E−06	+	8.92E−06	+	9.56E−06	+	2.86E−06	+	1.66E−05	+
F22	1.00E−01	−	1.51E−08	+	1.46E−05	+	4.06E−01	−	5.97E−06	+	1.12E−05	+	NA	−
F23	1.90E−09	+	5.83E−09	+	1.54E−05	+	4.51E−01	−	1.65E−05	+	1.56E−05	+	1.36E−05	+

**Table 13:** The IEEE CEC2022 test functions’ *p *values at the 5% significant level using the Wilcoxon rank- sum test.

Func	SCA vs EOBAVO		PSO vs EOBAVO		WOA vs EOBAVO		SSA vs EOBAVO		AVO vs EOBAVO		OBAVO vs EOBAVO		ROBAVO vs EOBAVO	
	*P *values	H	*P *values	H	*P *values	H	*P *values	H	*P *values	H	*P *values	H	*P *values	H
CEC01	4.27E−06	+	1.57E−05	+	1.11E−05	+	1.04E−05	+	5.73E−06	+	8.48E−05	+	3.89E−03	+
CEC02	1.14E−05	+	3.04E−06	+	8.76E−01	−	8.94E−01	−	1.38E−01	−	1.02E−04	+	1.51E−01	−
CEC03	1.27E−05	+	1.11E−06	+	6.01E−06	+	1.50E−05	+	1.85E−01	−	3.03E−06	+	6.43E−01	−
CEC04	9.98E−06	+	1.24E−05	+	1.47E−05	+	1.32E−05	+	1.42E−05	+	1.46E−04	+	9.11E−06	+
CEC05	7.90E−01	−	9.62E−06	+	6.58E−06	+	2.54E−06	+	2.14E−06	+	1.56E−04	+	1.52E−01	−
CEC06	6.90E−06	+	1.41E−05	+	1.3681E+00	−	5.22E−06	+	6.66E−06	+	9.47E−05	+	1.05E−01	−
CEC07	5.84E−01	−	2.34E−06	+	7.36E−01	−	4.16E−06	+	1.34E−05	+	1.17E−04	+	1.96E−01	−
CEC08	1.56E−05	+	1.33E−05	+	6.32E−06	+	1.25E−05	+	2.51E−01	−	1.55E−04	+	1.57E−05	+
CEC09	1.39E−05	+	3.38E−06	+	7.75E−01	−	5.62E−07	+	3.84E−06	+	1.18E−04	+	1.05E−05	+
CEC10	1.62E−05	+	2.74E−06	+	5.05E−06	+	9.55E−06	+	1.21E−05	+	8.56E−02	−	5.61E−01	−
CEC11	2.08E−06	+	2.75E−06	+	1.25E−05	+	1.28E−05	+	1.21E−05	+	9.66E−05	+	2.33E−01	−
CEC12	1.22E−05	+	1.36E−05	+	8.42E−06	+	1.47E−01	−	1.07E−05	+	1.02E−04	+	1.33E−05	+

It is clear from the aforementioned tables that the proposed EOBAVO, outperforms the other MAs because most of the *p* values are less than 0.05. Also, it must be noted that NA represents the statistical equivalence between the various algorithms.

### Convergence analysis

**Fig. 16:**
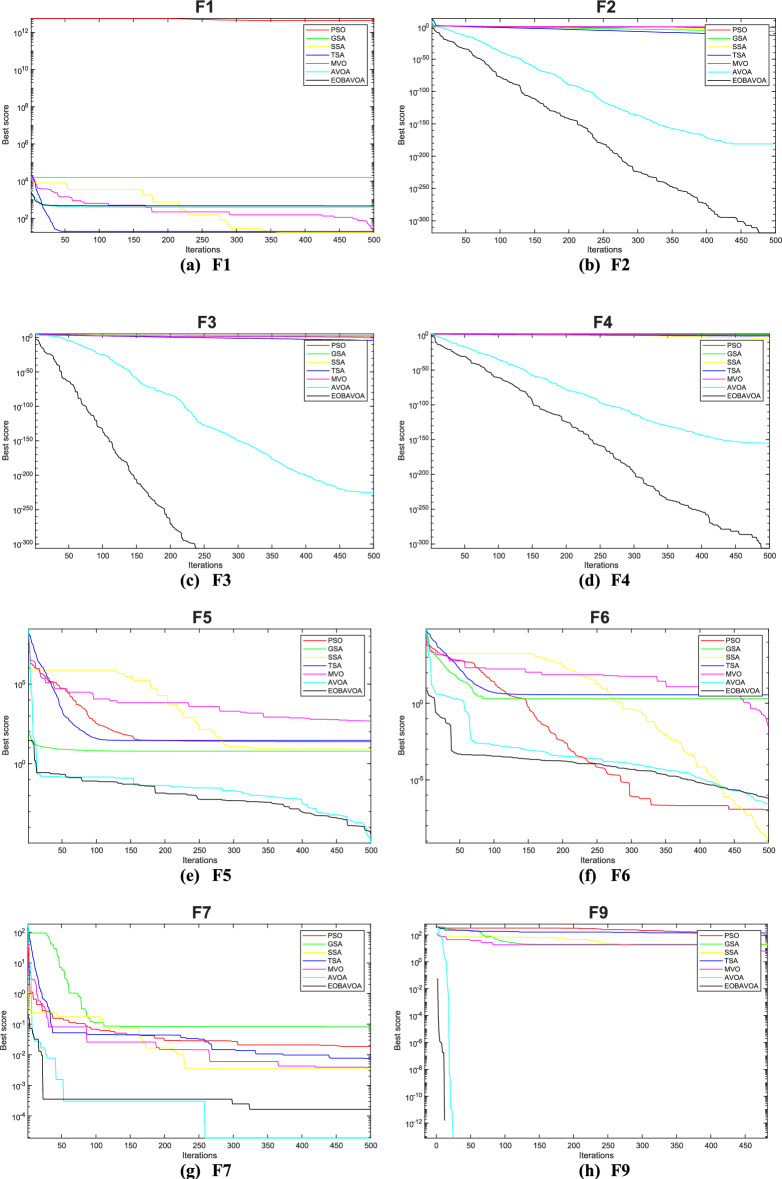

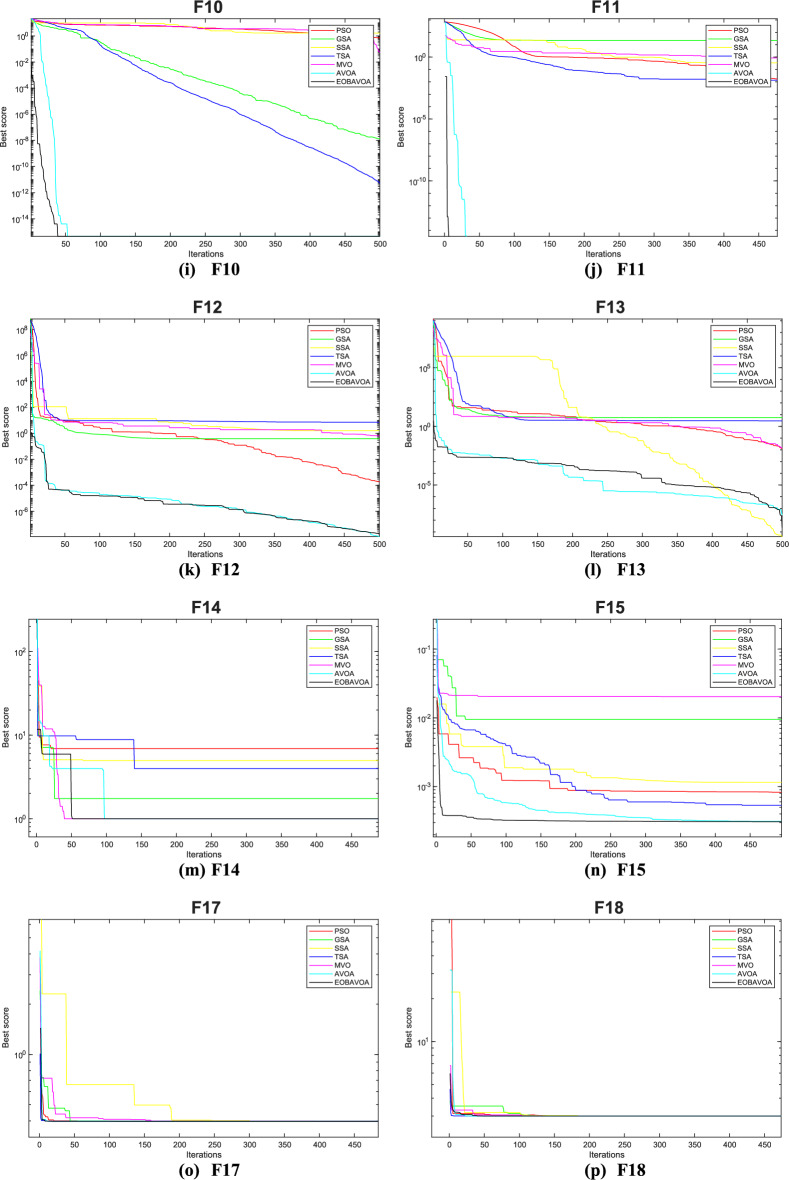
Convergence curves of EOBAVO and some cutting-edge algorithms used to solve the IEEECEC2005 test functions, respectively.

**Fig. 17:**
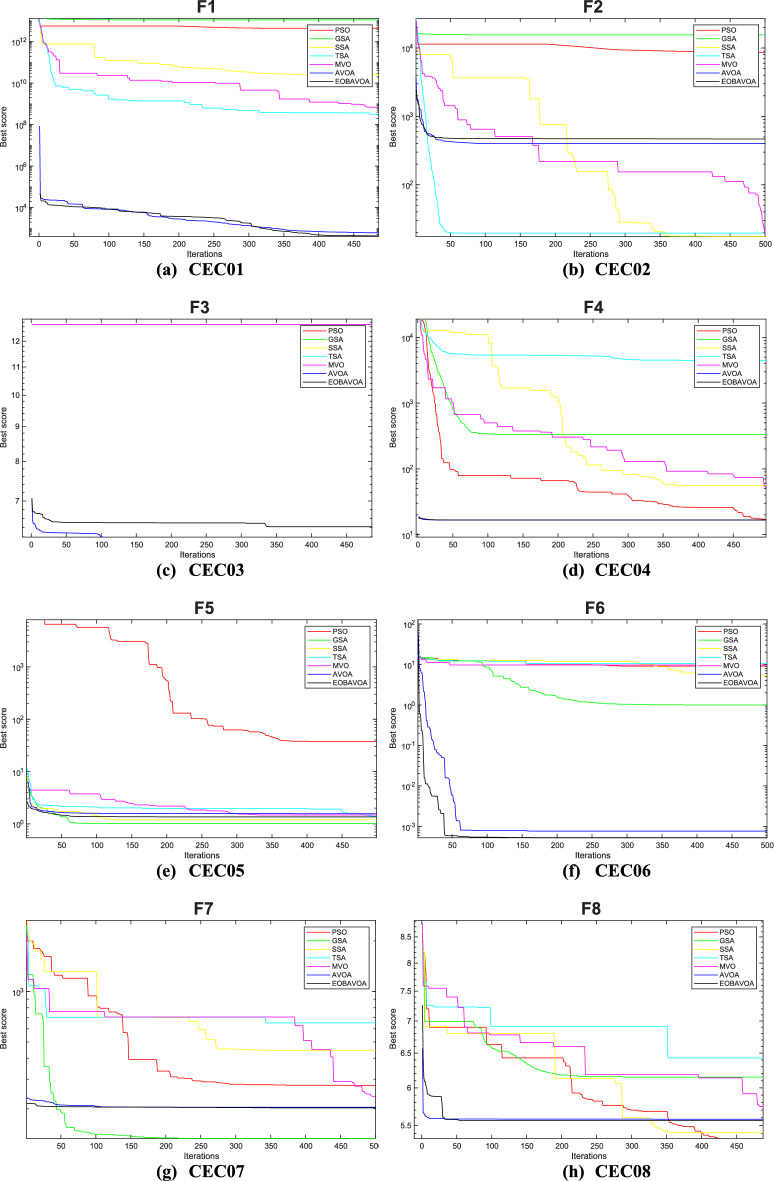

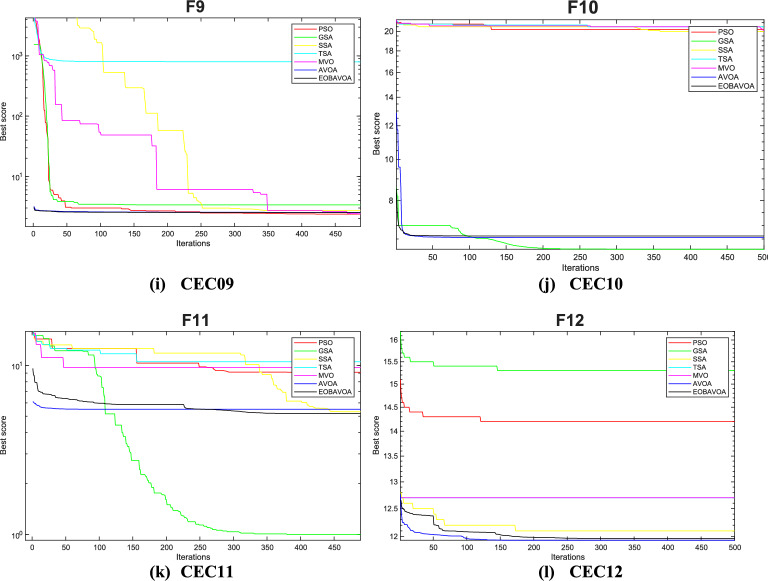
Convergence curves of EOBGAVO and some cutting-edge algorithms used to solve the IEEECEC2022 test functions, respectively.

The convergence curve illustrates the correlation between the number of iterations and the value of the fitness function. In the initial stages of optimization, the search agent quickly deviates from the designated search area. The primary objective of this convergence analysis is to examine the optimization behavior and graphical representation of the proposed EOBAVO technique. Figures 16 and 17 illustrate the convergence graphs for all the suggested algorithms that were evaluated against the IEEE CEC2005^65^ and IEE CEC2022^66^ test functions, respectively. Figure 16 shows that EOBAVO converges more quickly for all functions except F1 and F14 in unimodal functions, whereas the suggested approach performs best for functions F9, F10, and F11 in multimodal functions. The proposed method exhibits comparable convergence for functions F14, F15, F17, and F18, falling under the fixed-dimensional multi-modal category. Furthermore, when compared to the AVO, OBAVO, and ROBAVO methods, the EOBAVO approach has a greater impact on balancing convergence and divergence.

Additionally, it is clear from Fig. 17 that the EOBAVO technique outperforms all other algorithms in CEC01, CEC02, CEC03, CEC05, CEC08, and CEC10. However, compared to AVO, OBAVO, and ROBAVO, it is more successful at reaching convergence. Due to these improvements, the EOBAVO technique outperforms all the other algorithms under comparison both for convergence and search rate.

### EOBAVO’s comparison with the newest and most effective algorithms

This section compares the effectiveness of the proposed EOBAVO technique to the four most current and effective optimization algorithms: The Mountain Gazelle Optimization (MGO)^30^, Harris Hawks Optimization (HHO)^63^, the Hunger Games Search (HGS)^29^, and the weighted vectors INFO^64^. The IEEE CEC2005 and IEEE CEC2022 benchmark functions are used to implement the suggested method together with the most recent and effective algorithms.

Table 14 presents the statistical results of the IEEE CEC2005 test functions. The suggested EOBAVO technique performed better than all other equated algorithms and produced optimal solutions. According to the results in Table 14. For functions F1, F4, F6, and F15, EOBAVO produces better results; however, for functions F10, F20, and F23, the average fit values and standard deviation are roughly the same.

**Table 14:** The IEEE CEC2005 benchmark results use the newest and most effective methods.

Function		MGO	HHO	HGS	INFO	EOBAVO
F1	Mean	9.30E−71	1.01E−92	3.90E−167	1.09E−53	0.00E+00
	Std	5.03E−70	5.50E−92	0.00E+00	6.69E−54	0.00E+00
	t-values	− 1.01E+00	− 1.00E+00	N.A	− 8.91E+00	NA
F2	Mean	2.65E−41	2.33E−50	2.75E−76	1.13E−26	1.85E−256
	Std	1.35E−40	1.23E−49	1.50E−75	3.73E−27	0.00E+00
	t-values	− 1.07E+00	− 1.04E+00	− 1.00E+00	− 1.66E+01	NA
F3	Mean	2.57E−08	7.53E−75	2.12E−76	1.42E−50	0.00E+00
	Std	9.38E−08	3.30E−74	1.16E−75	1.16E−50	0.00E+00
	t-values	− 1.50E+00	− 1.25E+00	− 1.00E+00	− 6.66E+00	NA
F4	Mean	3.27E−24	5.86E−49	2.31E−65	1.78E−27	7.40E−263
	Std	1.12E−23	2.20E−48	1.27E−64	1.08E−27	0.00E+00
	t-values	− 1.60E+00	− 1.46E+00	− 1.00E+00	− 9.00E+00	NA
F5	Mean	1.06E−20	2.18E−02	1.17E+01	2.28E+01	3.75E−05
	Std	4.87E−20	3.07E−02	1.28E+01	6.37E−01	2.83E− 05
	t-values	7.24E+00	−3.88E+00	−5.04E+00	−1.96E+02	NA
F6	Mean	1.64E−08	1.51E−04	6.63E−06	6.06E−09	3.54E−07
	Std	5.25E−08	2.30E−04	1.10E−05	1.00E−08	3.27E−07
	t-values	5.59E+00	− 3.60E+00	− 3.11E+00	5.83E+00	NA
F7	Mean	3.13E−04	1.20E−04	1.70E−03	1.56E−03	1.15E−04
	Std	2.31E−04	1.39E−04	2.39E−03	1.20E−03	9.45E−05
	t-values	− 4.32E+00	− 1.34E−01	− 3.63E+00	− 6.59E+00	NA
F8	Mean	− 1.26E+04	− 1.24E+04	− 1.22E+04	− 8.88E+03	− 1.24E+04
	Std	1.02E−07	5.53E+02	1.07E+03	8.35E+02	3.44E+02
	t-values	2.90E+00	3.10E−01	−8.26E−01	−2.13E+01	NA
F9	Mean	0.00E+00	0.00E+00	0.00E+00	0.00E+00	0.00E+00
	Std	0.00E+00	0.00E+00	0.00E+00	0.00E+00	0.00E+00
	t- values	N. A	N. A	N. A	N. A	N. A
F10	Mean	1.04E−15	4.44E−16	4.44E−16	4.44E−16	4.44E−16
	Std	1.35E−15	1.50E−31	1.50E−31	1.50E−31	3.01E−31
	t-values	− 2.41E+00	8.83E+00	8.83E+00	8.83E+00	NA
F11	Mean	0.00E+00	0.00E+00	0.00E+00	0.00E+00	0.00E+00
	Std	0.00E+00	0.00E+00	0.00E+00	0.00E+00	0.00E+00
	t-values	N.A	N.A	N. A	N.A	NA
F12	Mean	6.09E−26	1.44E−05	1.19E−07	9.42E−10	1.94E−08
	Std	1.70E−25	2.15E−05	1.21E−07	2.01E−09	1.36E−08
	t-values	7.79E+00	− 3.67E+00	− 4.47E+00	7.34E+00	NA
F13	Mean	1.61E−32	1.44E−05	2.05E−02	5.75E−02	3.89E−08
	Std	4.35E−33	2.15E−05	6.83E−02	8.76E−02	3.30E−08
	t-values	6.45E+00	− 3.67E+00	− 1.64E+00	− 3.60E+00	NA
F14	Mean	9.98E−01	1.50E+00	1.98E+00	1.46E+00	1.13E+00
	Std	3.39E−16	9.97E−01	2.98E+00	9.29E−01	5.03E−01
	t-values	1.44E+00	− 1.79E+00	− 1.53E+00	− 1.71E+00	NA
F15	Mean	3.85E−04	3.38E−04	5.83E−04	1.23E−03	3.33E−04
	Std	2.42E−04	2.75E−05	2.53E−04	3.64E−03	8.33E−05
	t- values	− 1.10E+00	− 3.12E−01	− 5.14E+00	− 1.35E+00	NA
F16	Mean	− 1.03E+00	− 1.03E+00	− 1.03E+00	− 1.03E+00	− 1.03E+00
	Std	6.78E−16	2.94E−09	6.78E−16	6.78E−16	0.00E+00
	t- values	− 8.62E+01	− 2.16E+00	− 8.62E+01	− 8.62E+01	NA
F17	Mean	3.98E−01	3.98E−01	3.98E−01	3.98E−01	3.98E−01
	Std	1.13E−16	2.55E−05	1.13E−16	1.13E−16	1.13E−16
	t- values	0.00E+00	− 2.40E+00	0.00E+00	0.00E+00	NA
F18	Mean	3.00E+00	3.00E+00	3.00E+00	3.00E+00	3.00E+00
	Std	2.57E−15	1.84E−07	3.40E−15	3.20E−10	3.06E−06
	t-values	3.40E+00	3.19E+00	3.40E+00	3.40E+00	NA
F19	Mean	− 3.86E+00	− 3.86E+00	− 3.86E+00	− 3.86E+00	− 3.86E+00
	Std	1.36E−15	4.00E−03	1.36E−15	1.36E−15	2.53E−12
	t-values	1.28E+00	−4.30E+00	1.28E+00	1.28E+00	NA
F20	Mean	− 3.27E+00	− 3.10E+00	− 3.24E+00	− 3.27E+00	− 3.30E+00
	Std	5.99E−02	9.74E−02	7.95E−02	6.03E−02	4.51E−02
	t-values	− 2.32E+00	− 1.05E+01	− 3.90E+00	− 2.59E+00	NA
F21	Mean	− 1.02E+01	− 5.22E+00	− 9.47E+00	− 9.06E+00	− 1.02E+01
	Std	1.81E−15	9.01E−01	1.76E+00	2.53E+00	5.29E−13
	t-values	1.66E+00	− 3.00E+01	− 2.11E+00	− 2.36E+00	NA
F22	Mean	− 1.04E+01	− 5.08E+00	− 9.69E+00	− 8.40E+00	− 1.04E+01
	Std	9.03E−15	6.73E−03	1.84E+00	3.40E+00	3.20E−12
	t-values	1.24E+00	− 4.33E+03	− 2.11E+00	− 3.23E+00	NA
F23	Mean	− 1.05E+01	− 5.64E+00	− 1.04E+01	− 7.13E+00	− 1.05E+01
	Std	9.03E−15	1.60E+00	9.87E−01	3.77E+00	4.56E−13
	t-values	1.98E+00	− 1.67E+01	− 1.01E+00	− 4.95E+00	NA
w/t/l		4/12/7	13/7/3	10/11/2	14/5/4	

**Fig. 18:**
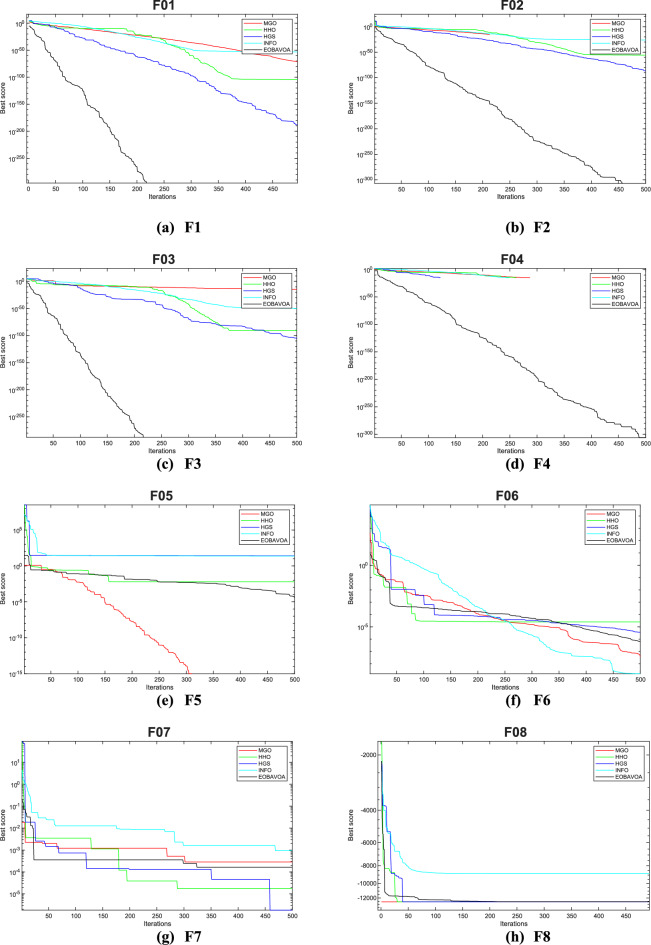

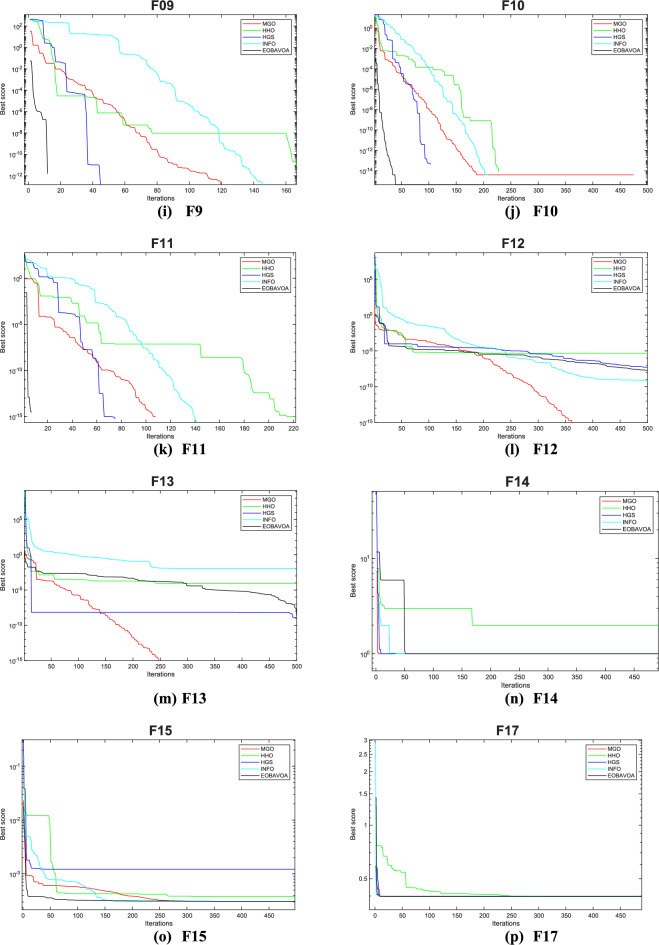

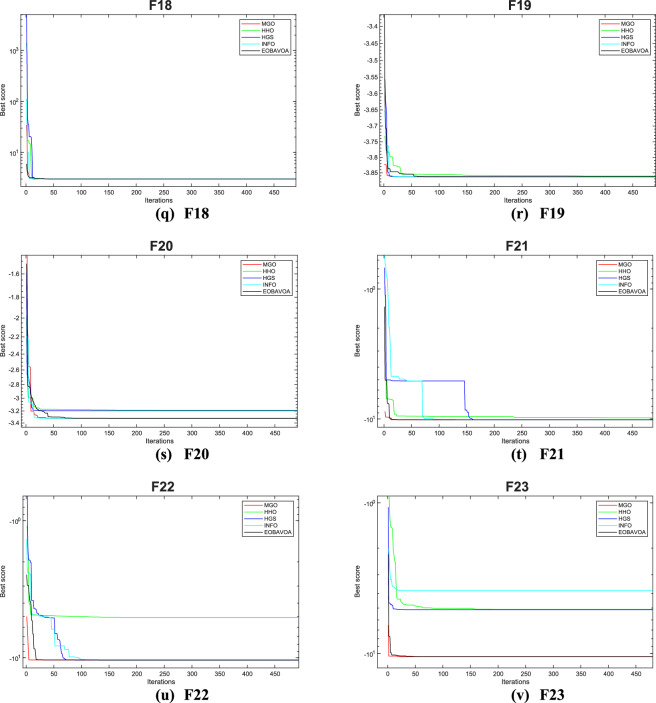
Convergence curves of the EOBAVO algorithm about the newest and most effective algorithms for solving the IEEECEC2005 benchmark functions.

Table 15 shows the results of optimization and significance testing for the IEEE CEC2022 benchmark functions, using EOBAVO and the latest and most effective methodologies. The suggested method outperformed the others for functions F1, F3, F8, and F10, as Table 15 demonstrates. As a result, the suggested method outperforms the most recent algorithms, MGO, HHO, HGS, and INFO. These algorithms’ convergence curves have been run and are shown in Fig. 18. The analysis of these curves indicates that the EOBAVO method demonstrates superior performance across the majority of functions. Almost all of the *p* values in Table 15 fall below a 5% significance level, indicating that the recommended method demonstrates better performance compared to the other algorithms.

**Table 15:** The IEEE CEC2022 benchmark function’s results using the newest and most effective methods.

Func		MGO	HHO	HGS	INFO	EOBAVO
CEC01	Mean	3.00E+02	1.01E+03	3.75E+02	3.00E+02	0.00E+00
	Std	1.18E−04	3.85E+02	2.97E+02	5.58E−06	0.00E+00
	t-values	2.67E+00	−2.46E+00	1.92E+00	2.67E+00	NA
CEC02	Mean	4.07E+02	4.67E+02	4.25E+02	4.09E+02	1.85E−256
	Std	1.25E+01	8.61E+01	3.16E+01	1.20E+01	0.00E+00
	t-values	2.99E+00	−2.39E+00	1.62E−01	2.80E+00	NA
CEC03	Mean	6.00E+02	6.36E+02	6.01E+02	6.01E+02	0.00E+00
	Std	5.31E−01	1.11E+01	1.04E+00	2.64E+00	0.00E+00
	t-values	1.10E+01	−4.94E+00	1.07E+01	1.01E+01	NA
CEC04	Mean	8.13E+02	8.28E+02	8.37E+02	8.21E+02	7.40E−263
	Std	4.84E+00	7.16E+00	1.44E+01	8.61E+00	0.00E+00
	t-values	1.16E+01	6.42E−01	−2.88E+00	4.24E+00	NA
CEC05	Mean	9.04E+02	1.41E+03	1.17E+03	9.30E+02	3.75E−05
	Std	7.26E+00	1.74E+02	2.25E+02	4.00E+01	2.83E−05
	t-values	1.67E+01	−2.16E+00	3.29E+00	1.50E+01	NA
CEC06	Mean	2.20E+03	8.19E+03	4.35E+03	2.49E+03	3.54E−07
	Std	4.16E+02	5.12E+03	2.35E+03	1.50E+03	3.27E−07
	t-values	3.65E+00	−5.15E+00	−2.30E+00	1.87E+00	NA
CEC07	Mean	2.02E+03	2.08E+03	2.02E+03	2.02E+03	1.15E−04
	Std	6.70E+00	3.08E+01	5.16E+00	7.62E+00	9.45E−05
	t-values	5.01E+00	−5.24E+00	5.82E+00	4.82E+00	NA
CEC08	Mean	2.22E+03	2.23E+03	2.21E+03	2.22E+03	−1.24E+04
	Std	4.78E+00	2.27E+01	3.50E+00	5.67E+00	3.44E+02
	t-values	4.37E+00	−2.23E+00	2.60E+00	4.50E+00	NA
CEC09	Mean	2.53E+03	2.61E+03	2.54E+03	2.53E+03	0.00E+00
	Std	4.63E−13	3.73E+01	3.00E+01	2.11E−12	0.00E+00
	t-values	2.52E+00	−6.52E+00	1.16E+00	2.52E+00	NA
CEC10	Mean	2.55E+03	2.58E+03	2.56E+03	2.57E+03	4.44E−16
	Std	5.80E+01	1.05E+02	7.51E+01	6.84E+01	3.01E−31
	t-values	1.05E+00	−6.34E−01	4.43E−01	8.25E−02	NA
CEC11	Mean	2.75E+03	2.76E+03	2.76E+03	2.75E+03	0.00E+00
	Std	1.30E+02	1.40E+02	1.45E+02	1.43E+02	0.00E+00
	t-values	−1.35E+00	−1.82E+00	−1.76E+00	−9.60E−01	NA
CEC12	Mean	2.86E+03	2.92E+03	2.87E+03	2.86E+03	1.94E−08
	Std	2.28E+00	5.52E+01	4.19E+00	2.02E+00	1.36E−08
	t-values	1/1/10	11/0/1	3/2/7	1/0/11	NA

Therefore, the statistical findings of the IEEE CEC2005 and IEEE CEC2022 research show that the EOBAVO outperforms the most recent and effective technique.

Figure 18 shows the convergence graphs of the EOBAVO technique in relation to the most recent and effective algorithms. The statistical results demonstrated that the proposed EOBAVO technique performed similarly to MGO and HGS but better than HHO, INFO.

**Fig. 19:**
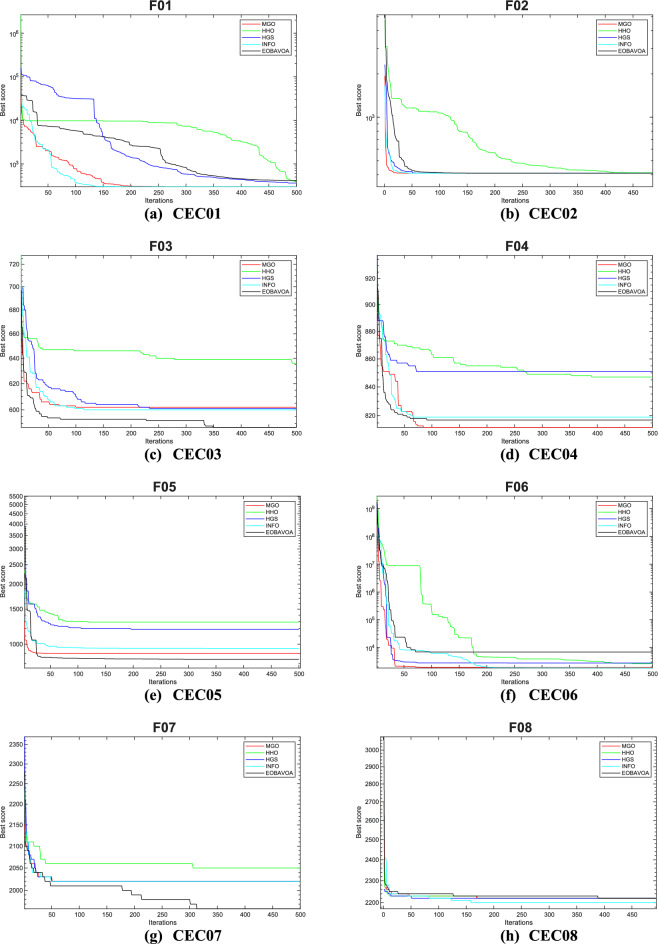

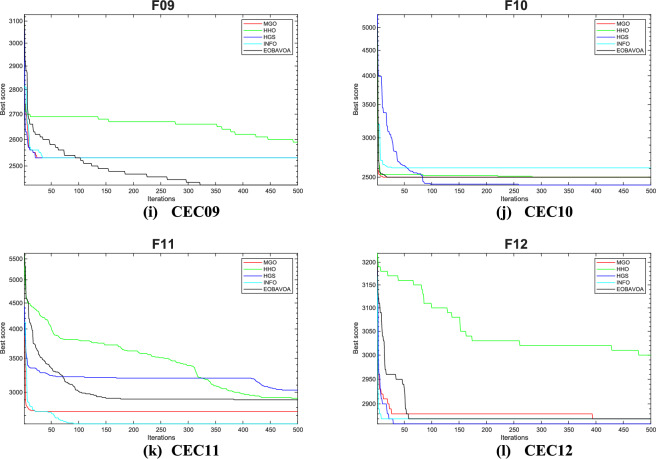
Convergence curves of the EOBAVO algorithm with the newest and most effective method for solving the benchmark functions in IEEECEC2022**.**

These algorithms’ convergence curves have been run and are shown in Fig. 19. It clearly shows that the EOBAVO method works better for most functions.

EOBAVO method performs better than the other methods, as shown by the majority of the *p* values in Tables 16 and 17 being below the 5% significance level. Therefore, the statistical findings of the IEEE CEC2005 and IEEE CEC2022 examinations show that the EOBAVO outperforms the most recent and effective technique.

**Table 16:** Results of the Wilcoxon rank-sum test for the IEEECEC2005 test functions, at a significance level of 5%

Func	HHO vs EOBAVO		MGO vs EOBAVO		HGS vs EOBAVO		INFO vs EOBAVO	
	*P *values	H	*P *values	H	*P *values	H	*P *values	H
F1	1.65E−06	+	1.36E−06	+	1.38E−06	+	2.66E−01	−
F2	1.98E−06	+	NA	−	7.85E−07	+	1.30E−06	+
F3	1.20E−06	+	NA	−	2.78E−06	+	6.93E−01	−
F4	1.38E−06	+	1.41E−06	+	2.81E−06	+	1.70E−06	+
F5	5.41E−01	−	1.04E−06	+	1.56E−06	+	1.54E−07	+
F6	1.48E−06	+	1.76E−07	+	1.63E−06	+	1.86E−06	+
F7	1.72E−06	+	1.97E−08	+	1.79E−06	+	1.25E−06	+
F8	1.83E−06	+	1.90E−06	+	3.16E−06	+	2.13E−01	−
F9	2.11E−06	+	1.80E−06	+	NA	−	1.50E−01	−
10	1.50E−06	+	1.97E−07	+	NA	−	3.46E−01	−
F11	1.46E−06	+	1.15E−06	+	NA	−	1.78E−06	+
F12	1.15E−06	+	1.55E−07	+	2.25E−06	+	1.06E−06	+
F13	7.80E−01	−	1.29E−06	+	3.08E−06	+	1.05E−06	+
F14	2.06E−01	−	1.98E−06	+	1.15E−07	+	1.64E−06	+
F15	1.99E−07	+	7.15E−01	−	2.04E−01	−	1.38E−06	+
F16	1.75E−06	+	1.10E−06	+	1.48E−01	−	5.34E−01	−
F17	1.17E−07	+	1.11E−06	+	1.46E−01	−	2.68E−01	−
F18	1.24E−06	+	1.96E−06	+	1.87E−06	+	1.78E−06	+
F19	1.30E−06	+	1.63E−01	−	1.14E−06	+	5.03E−01	−
F20	1.04E−06	+	8.53E−01	−	4.32E−07	+	1.03E−06	+
F21	1.32E−07	+	2.99E−01	−	8.95E−08	+	1.77E−06	+
F22	1.45E−07	+	1.03E−06	+	2.63E−01	−	1.30E−07	+
F23	1.51E−06	+	1.16E−06	+	1.04E−01	−	1.96E−06	+

**Table 17:** Results of the Wilcoxon rank-sum test for the IEEECEC2022 test functions, at a significance level of 5%.

Function	EOBAVO vs HHO		EOBAVO vs MGO		EOBAVO vs HGS		EOBAVO vs INFO	
	*P* value	H	*P* value	H	*P* value	H	*P* value	H
CEC01	1.73E−07	+	1.00E−01	−	1.16E−07	+	1.02E−06	+
CEC02	1.83E−07	+	NA	−	7.43E+00	−	1.02E−06	+
CEC03	1.38E−06	+	NA	−	1.78E−06	+	1.83E−07	+
CEC04	1.57E−06	+	1.30E−07	+	1.00E−07	+	1.76E−01	−
CEC05	1.58E−06	+	1.10E−06	+	1.58E−07	+	2.07E−01	−
CEC06	NA	−	1.76E−06	+	1.26E−07	+	1.64E−01	−
CEC07	NA	−	1.60E−07	+	1.23E−06	+	6.08E−01	−
CEC08	1.88E−06	+	1.55E−06	+	1.10E−06	+	1.73E−07	+
CEC09	1.53E−06	+	1.64E−06	+	1.96E−07	+	1.13E−06	+
CEC10	1.87E−07	+	1.18E−06	+	1.63E−06	+	1.18E−06	+
CEC11	1.94E−06	+	1.51E−06	+	1.73E−07	+	1.78E−06	+
CEC12	1.41E−06	+	1.34E−06	+	1.91E−06	+	1.51E−06	+

### Exploration and exploitation analysis

**Fig. 20:**
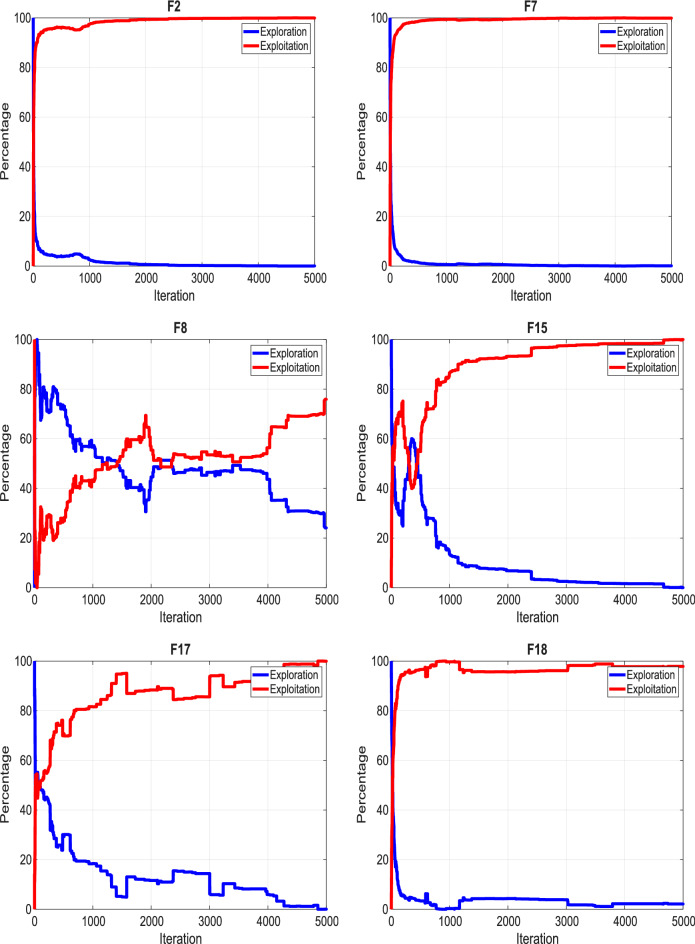
Exploration/exploitation analysis of EOBAVO.

To assess the exploration and exploitation capabilities of EOBAVO, the average Euclidean distance from each individual to the best-known solution throughout iterations was monitored. Higher average distances are indicative of exploration, while lower distances indicate exploitation^46,47^. Examining the balance between the exploration and exploitation phases of the EOBAVO can provide significant insights for tackling practical optimization issues. Figure 20 shows a graphical representation of the exploration and exploitation trajectories in the search space while addressing the difficulties associated with the CEC2005 and CEC2022 functions. It is essential to highlight that Functions F2. F7 and F8 represent the functions within CEC2022, while F15, F17 and F18 denote the benchmarks within CEC2005. The graphs show that during the early stages of the optimization, EOBAVO maintains a consistent pattern with high exploration, which rapidly decreases within the first few hundred iterations. Simultaneously, exploitation increases sharply and dominates the search process for the remainder of the optimization run. This shows with an effective convergence dynamic, EOBAVO is prompt in recognizing promising regions quickly. In contrast, F8 exhibits unstable exploration–exploitation dynamics, with persistent fluctuations and multiple crossovers throughout the run. This indicates a complex, multimodal landscape where the optimizer struggles to converge, suggesting the need for enhanced diversity or adaptive control mechanism. F15 also shows unstable fluctuations between exploration and exploitation throughout the iterations, indication inconsistent search behavior. This suggests that algorithm struggles to maintain focus, likely due to a highly multimodal or deceptive landscape. Exploration remains dominant for most of the iterations in F17, while exploitation stays low and erratic. This prolonged exploration phase reflects difficulty in converging and suggests potential improvements in the algorithm’s exploitation strategy. The algorithm quickly transitions from exploration to exploitation in F18, within the first 1000 iterations. This smooth shift indicates a well-balanced search process and efficient convergence toward optimal solutions. We can conclude that EOBAVO effectively transitions from exploration to exploitation and converges well on most functions, but struggles to maintain stability and achieve to maintain stability and achieve convergence on complex, multimodal landscapes such as F8, F15 and F17. These challenges highlight the need to enhance diversity management and introduce adaptive control mechanisms for improved performance.

#### Diversity analysis

In MA optimization, diversity analysis evaluates the distribution and speed of candidate solutions within the search space. It plays a critical role in understanding the exploration and exploitation balance of an algorithm. High diversity typically supports better global exploration, helping to avoid premature convergence to local optima, whereas low diversity aids in intensifying the search near promising regions for convergence^47^. There are nonlinear features in the diversity trajectory. The average distance travelled by each person during the iterative process is represented by the trajectory^72^. Greater aggregation within the population throughout the optimization process is indicated by less nonlinear population diversity, and this raises the possibility of convergence towards a sub-optimal solution within the problem space. The greatest separation between two members of the population was determined to be the population’s radius. Too much diversity can hinder convergence, while too little may lead to stagnation. The population radius is the maximum distance between any two individuals within the population and mathematically represented by Eq. (25).

25 $$\begin{array}{*{20}c} {\left| D \right| = \mathop {\max }\limits_{{\left( {i \ne j} \right) \in \left| {1,\left| S \right|} \right|}} \left( {\sqrt {\mathop \sum \limits_{t = 1}^{Dim} \left( {x_{it} - x_{jt} } \right)^{2} } } \right) } \\ \end{array}$$

where the $\left| S \right|$ and *Dim* represents the population size and dimension of the problem size respectively, $x_{it}$ denotes the position in the ith dimension of the ith individual. Finally, the diversity population from Eq. (25) is given by Eq. (26) as follows:

26 $$\begin{array}{*{20}c} {D^{N} = \frac{1}{\left| S \right|\left| D \right|}\mathop \sum \limits_{I = 1}^{\left| S \right|} \sqrt {\mathop \sum \limits_{t = 1}^{Dim} \left( {x_{it} - \overline{x}_{t} } \right)^{2} } } \\ \end{array}$$

**Fig. 21:**
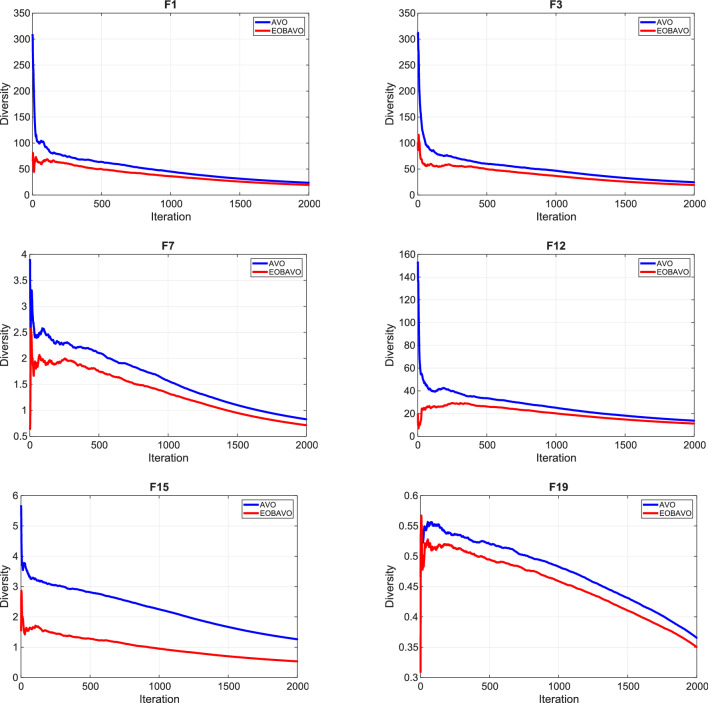
Diversity graphs of AVO and EOBAVO.

And the ${\overline{x} }_{t}$ is the center of the populace. The diversity analysis of selected benchmark functions from CEC2005 and CEC2022 is represented in Fig. 21. We selected F1, F15 and F19 to represent CEC2005, while CEC2022 is represented by F3, F7 and F12. From the graphs, EOBAVO consistently maintained lower diversity levels for CEC2005 functions, particularly in F15 and F19. This means that with a reduced diversity, EOBAVO exploited the search space more aggressively, enabling faster convergence but at the potential risk of reduced exploration. In F1, both algorithms showed a similar decline in diversity, though AVO retained higher diversity for a longer period, suggesting greater exploration capacity in simpler unimodal landscapes. In contrast, the diversity gap between AVO and EOBAVO widened in F15 and F19, highlighting EOBAVO’s stronger exploitation tendencies on more complex multimodal functions. On the CEC2022 functions, EOBAVO consistently exhibited lower diversity than AVO, with the largest difference in F7. This indicates that EOBAVO’s enhanced OBL intensifies exploitation and accelerates convergence, especially in complex landscapes. However, the reduced diversity may limit exploration, while AVO maintains broader search capability at the cost of slower convergence. Overall, EOBAVO enhanced convergence speed by reducing population diversity and promoting exploitation across benchmarks.

## Performance of EOBAVO on practical engineering problems

In this section, the proposed EOBAVO technique is examined for its performance and effectiveness by applying it to various real-world engineering design issues^73^. These issues encompass the pressure vessels, the speed reducers, the welded beams, the tension/compression spring, and the gear train problems.

The EOBAVO method is employed to address each issue, and the outcomes are compared with those obtained from other advanced algorithms such as AVO, OBAVO, ROBAVO, PSO, WOA, SCA, and SSA.

### Pressure vessel problems

**Fig. 22:**
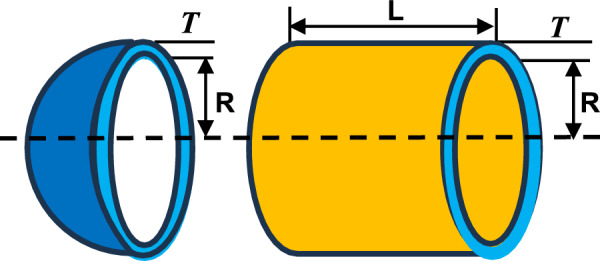
Pressure vessel problems.

The main aim of this engineering challenge is to reduce the total cost, which includes the material, welding, and forming, of a cylindrical vessel. Figure 22 shows the vessel’s schematic representation. The shell’s width (*T*_*s*_), the width of the head (*T*_*h*_), the cylinder’s length without measuring the radius (*R*), and the head (*L*) are the four decision parameters in this pressure vessel problem.

The design can be stated mathematically as

$$\vec{p} = \left[ {p_{11} , p_{22} , p_{33} , p_{44} } \right] = \left[ {T_{s} T_{h} RL} \right]$$

27 $$\begin{array}{*{20}c} {Minimize f\left( {\vec{p}} \right) = 0.6224 p_{11} p_{33} p_{44} + 1.7781p_{22} p_{33}^{2} + 3.1661p_{11}^{2} p_{44} + 19.84p_{11}^{2} p_{33} } \\ \end{array}$$

Subject to

$$\begin{aligned} g_{1} \left( {\vec{p}} \right) & = - p_{11} + 0.0193p_{33} \le 0, \\ g_{2} \left( {\vec{p}} \right) & = - p_{22} + 0.00954p_{33} \le 0, \\ g_{3} \left( {\vec{p}} \right) & = - \pi p_{33}^{2} p_{44} - \frac{4}{3}\pi p_{33}^{3} + 1296000 \le 0, \\ g_{4} \left( {\vec{p}} \right) & = p_{44} - 240 \le 0, \\ \end{aligned}$$

The following are the decision variables’ ranges:

$$0 \le p_{11} \le 99,0 \le p_{22} \le 99,10 \le p_{33} \le 200,10 \le p_{44} \le 200$$

The records of the aforementioned MAs are compared with the results of the EOBAVO approach. The best results from each algorithm are compared in Table 18, showing that the EOBAVO method minimizes the costs of the cylindrical pressure vessel with superior performance.

**Table 18:** Outcomes of the pressure vessel problem in EOBAVO.

Algorithms	Variable optimal values				Optimal costs
	T_s_	T_h_	R	L	
EOBAVO	4.010E+01	1.394E+01	1.016E+01	6.113E+01	**3.797E+05**
ROBAVO	6.241E+01	4.577E+01	1.417E+02	5.242E+01	1.322E+07
OBAVO	9.521E+00	4.400E+00	1.348E+02	1.407E+02	5.056E+05
AVO	8.349E+01	2.839E+01	9.430E+01	7.632E+01	1.406E+07
PSO	6.000E+01	1.757E+01	4.114E+01	1.283E+01	3.024E+06
SSA	5.082E+01	4.869E+01	1.694E+02	6.216E+01	1.197E+07
SCA	5.077E+01	8.582E+01	5.344E+01	1.218E+01	3.473E+06
WOA	2.922E+01	8.265E+01	4.119E+01	6.141E+01	2.321E+06

### Problems involving welded beam design

**Fig. 23:**
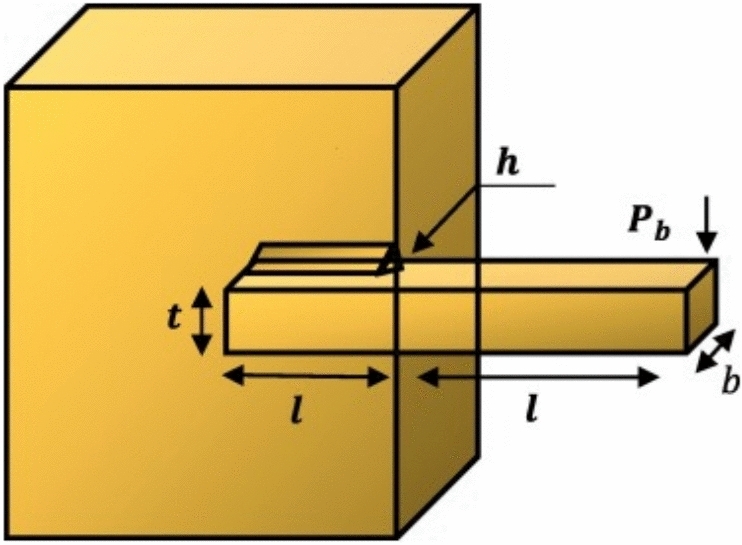
A Welded beam^73^.

The purpose of this problem is to create a beam that is economically viable in terms of production costs. Figure 20 shows the features and structure of the welded beam. The beam’s shear stress ($\tau$), bending stress ($\sigma$), buckling load (Pb) and the end deflection ($\delta$) are the optimization constraints in this issue. Additionally, there are four consecutive decision variables: Weld thickness (h), bar height (t), bar thickness (b), and clamped bar length (l). Figure 23 and Table 19 shows the welded beam and the outcome of the Welded Beam design in EOBAVO respectively.

**Table 19:** Outcomes of the welded beam design in EOBAVO.

Algorithms	Variable optimal values				Optimal costs
	h	l	t	b	
EOBAVO	7.0230E−01	2.9840E+00	4.5520E−01	1.8010E−01	**1.6929E+00**
ROBAVO	1.4236E+00	2.3872E+00	3.6850E−01	1.6930E+00	5.8363E+00
OBAVO	6.9270E−01	3.6289E+00	8.0755E+00	1.9479E+00	1.5262E+01
AVO	1.5529E+00	4.0545E+00	8.0915E+00	5.0410E−01	1.4343E+01
PSO	1.8938E+00	6.3116E+00	9.2170E+00	1.7938E+00	4.1159E+01
SSA	1.2563E+00	2.1049E+00	5.2850E−01	1.8264E+00	4.4177E+00
SCA	6.8970E−01	6.7768E+00	1.8051E+00	7.3400E−01	4.8852E+00
WOA	1.6302E+00	5.6260E+00	5.5820E−01	7.4400E−01	1.6909E+01

This problem can be represented numerically as follows:

28 $$\begin{array}{*{20}c} \begin{aligned} & \vec{w} = \left[ {w_{1} , w_{2} , w_{3} , w_{4} } \right] = \left[ {hltb} \right] \\ & Minimize\;f\left( {\vec{w}} \right) = 1.1047w_{1}^{2} w_{2} + 0.04811w_{3} w_{4} \left( {14.0 + w_{2} } \right) \\ \end{aligned} \\ \end{array}$$

$$\begin{aligned} {\text{Subject to}} & \\ & g_{1} \left( {\vec{w}} \right) = \tau \left( {\vec{w}} \right) - \tau _{{max}} \le 0, \\ & g_{2} \left( {\vec{w}} \right) = \sigma \left( {\vec{w}} \right) - \sigma _{{max}} \le 0 \\ & g_{3} \left( {\vec{w}} \right) = \delta \left( {\vec{w}} \right) - \delta _{{max}} \le 0 \\ & g_{4} \left( {\vec{w}} \right) = w_{1} - w_{4} \le 0 \\ & g_{5} \left( {\vec{w}} \right) = P - P_{c} \left( {\vec{w}} \right) \le 0, \\ & g_{6} \left( {\vec{w}} \right) = 0.125 - w_{1} \le 0, \\ & g_{7} \left( {\vec{w}} \right) = 1.1.471w_{1}^{2} + 0.04811w_{3} w_{4} \left( {14 + w_{2} } \right) - 5 \le 0 \\ \end{aligned}$$

The following are the decision variables’ ranges:

$$0.1 \le w_{1} \le 2,0.1 \le w_{2} \le 10,0.1 \le w_{3} \le 10,0.1 \le w_{4} \le 2$$

where

$$\tau \left( {\vec{w}} \right) = \sqrt {\left( {\tau \prime } \right)^{2} + 2\tau \prime \tau \prime \prime \frac{{w_{2} }}{2R} + \left( {\tau \prime ^{\prime}} \right)^{2} } ,\quad \tau \prime = \frac{P}{{\sqrt {2w_{1} w_{2} } }},\tau \prime \prime = \frac{MR}{J},$$

$$M = P\left( {L + \frac{{w_{2} }}{2}} \right), R = \sqrt {\frac{{w_{2}^{2} }}{4} + \left( {\frac{{w_{1} + w_{3} }}{2}} \right)^{2} }$$

$$J = 2\left\{ {\sqrt {2w_{1} w_{2} } \left[ {R^{2} } \right]} \right\},$$

$$\sigma \left( {\vec{w}} \right) = \frac{6PL}{{w_{4} w_{3}^{2} }}, \delta \left( {\vec{w}} \right) = \frac{{6PL^{3} }}{{Ew_{3}^{2} w_{4} }}$$

$$P_{c} \left( {\vec{w}} \right) = \frac{{4.013E\sqrt {\frac{{w_{3}^{2} w_{4}^{6} }}{36}} }}{{L^{2} }} \left( {1 - \frac{{w_{3} }}{2L} \sqrt{\frac{E}{4G}} } \right)$$

$$P = 6000lb, L = 14in., E = 30*10^{6} psi., G = 12*10^{6} psi$$

$$\tau_{max} = 13,600psi., \sigma_{max} = 30,000psi., \delta_{max} = 0.25in.$$

### Tension/compression spring design problems

**Fig. 24:**
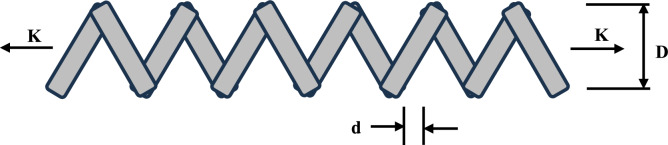
Compression/tension spring.

This engineering design problem’s primary objective was to reduce the weight of the spring, while three nonlinear inequality constraints were accounted for with a linear constraint. The wire diameter (d), the count of active coils (K), and the mean coil diameter (D) represent the three subsequent criteria for decision making in this engineering design. Figure 24 shows the spring’s schematic representation. The design’s numerical expression is shown as follows.

$$X = \left[ {x_{1} ,x_{2} ,x_{3} } \right] = \left[ {d,D,K} \right]$$

29 $$\begin{array}{*{20}c} {Minimize f\left( x \right) = \left( {x_{3} + 2} \right)x_{2} x_{1}^{2} } \\ \end{array}$$

$$\begin{aligned} & g_{1} \left( x \right) = 1 - \frac{{x_{2}^{3} x_{3} }}{{71.785x_{1}^{4} }} \le 0,\quad g_{2} \left( x \right) = \frac{{4x_{2}^{3} - x_{1} x_{2} }}{{12.566\left( {x_{2} x_{1}^{3} } \right)}} + \frac{1}{{5108x_{1}^{2} }} - 1 \le 0 \\ & g_{3} \left( x \right) = 1 - \frac{{140.45 x_{1} }}{{x_{2}^{2} x_{3} }} \le 0,\quad g_{4} \left( x \right) = \frac{{x_{1} + x_{2} }}{1.5} - 1 \le 0 \\ & 0.05 \le x_{1} \le 2,\quad 0.25 \le x_{2} \le 1.3\quad and\quad 2 \le x_{3} \le 15, \\ \end{aligned}$$

The results of the aforementioned MAs that have been successful in resolving this issue are compared to the results of the recently introduced EOBAVO approach. Table 20 presents the comparative findings, where, in terms of effectiveness, EOBAVO performs better than the other algorithms.

**Table 20:** EOBAVO’s findings for the compression or tension spring problem**.**

Algorithm	Variables optimal values			Optimum weight
	d	D	K	
EOBAVO	3.051E−01	3.600E−01	1.195E+01	**4.675E**−**01**
ROBAVO	1.640E+00	5.977E−01	4.265E+00	1.007E+01
OBAVO	3.917E−01	1.034E+00	1.048E+01	1.981E+00
AVO	8.888E−01	8.942E−01	1.389E+01	1.122E+01
PSO	8.088E−01	9.151E−01	1.007E+01	7.223E+00
SSA	1.630E+00	1.277E+00	2.581E+00	1.553E+01
SCA	6.114E−01	6.769E−01	1.487E+01	4.270E+00
WOA	1.650E+00	6.538E−01	6.946E+00	**1.592E+01**

### Gear train design problems

**Fig. 25:**
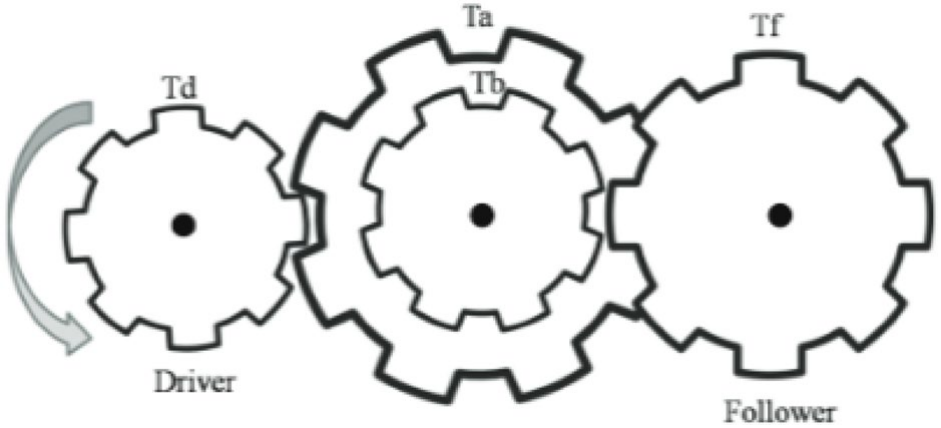
Gear train design^74^.

The key focus of a gear train design is the reduction of the gear ratio, which constitutes a mechanical problem. Figure 25 shows the schematic representation of a trained gear. Four successive decision variables with this engineering design include: ${n}_{A}\left({g}_{1}\right)$, ${n}_{B}\left({g}_{2}\right)$, ${n}_{C}\left({g}_{3}\right)$ and ${n}_{D}\left({g}_{4}\right)$.

This design’s mathematical expression has been provided in Eq. (30).

Let $$\vec{x} = \left[ {x_{1} , x_{2} , x_{3} , x_{4} } \right]$$ = $$n_{A} n_{B} n_{C} n_{D}$$

30 $$\begin{array}{*{20}c} {Minimize f\left( {\vec{x}} \right) = \left( {\frac{1}{6.931} - \frac{{x_{3} x_{2} }}{{x_{1} x_{4} }}} \right)^{2} } \\ \end{array}$$

The following are the design variables’ ranges:

$$x_{1} , x_{2} , x_{3} , x_{4} \in \left\{ {12, 13, 14, \ldots , 60} \right\}$$

The EOBAVO technique’s best results are compared to those of other MAs, and the results that are best from each algorithm are compared in Table 21, showing that the EOBAVO method fared better than the others.

**Table 21:** EOBAVO gear train trouble outcomes.

Algorithm	Variables optimal values				Optimum cost
	${n}_{A}$	${n}_{B}$	${n}_{C}$	${n}_{D}$	
EOBAVO	4.294E+01	2.166E+01	1.247E+01	2.207E+01	**1.977E**−**02**
ROBAVO	4.443E+01	1.366E+01	3.414E+01	2.999E+01	4.228E−02
OBAVO	4.369E+01	1.560E+01	3.250E+01	2.591E+01	9.225E−02
AVO	5.327E+01	1.567E+01	3.115E+01	2.915E+01	2.892E−02
PSO	4.108E+01	1.754E+01	4.586E+01	2.709E+01	3.346E−01
SSA	4.183E+01	2.264E+01	3.448E+01	2.814E+01	2.694E−01
SCA	5.605E+01	1.559E+01	3.930E+01	2.641E+01	7.276E−02
WOA	4.922E+01	1.862E+01	9.959E+01	2.972E+01	1.261E+00

### Speed reducer design problems

**Fig. 26:**
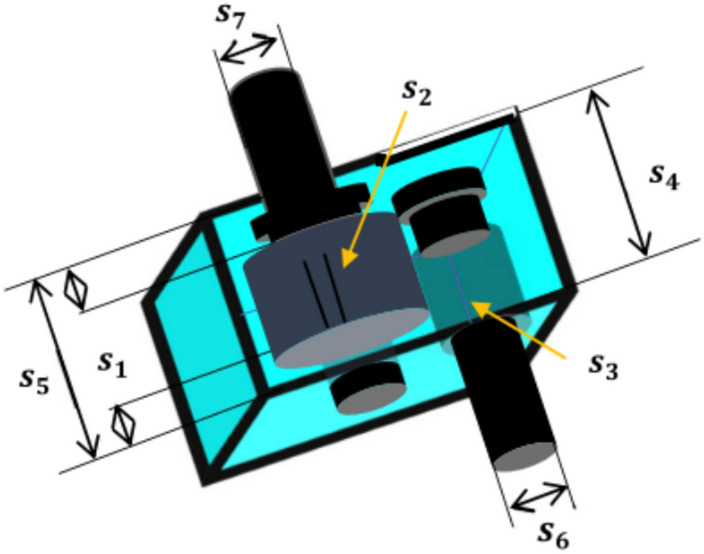
Speed reducer design problems^73^.

Designing a gear box speed reduction having the least amount of weight is the primary objective of this project. Eleven inequality constraints form the basis of the speed reducer weight; seven of these constraints are nonlinear, while the remaining four are linear. In this engineering problem, there are seven variables to consider: the module of teeth m(s_2_), the face width b(s_1_), the teeth count in the pinion z(s_3_), the first shaft’s length between bearings l_1_(s_4_), the first shaft’s diameter d_1_(s_6_), the second shaft’s length between the bearings l_2_(s_5_) and the second shaft’s diameter d_2_(s_7_). Figure 26 shows a graphic illustration of this design.

Mathematically, we can represent the design in Eq. (31):

$${\text{Suppose}}\;\vec{s} = \left[ {s_{1} ,s_{2} ,s_{3} ,s_{4} ,s_{5} ,s_{6} ,s_{7} } \right] = \left[ {bmxl_{1} l_{2} d_{1} d_{2} } \right]$$

31 $$\begin{array}{*{20}c} {Minimize f\left( {\vec{s}} \right) = 0.7854s_{1} s_{2}^{2} \left( {3.333s_{3}^{2} + 14.9334s_{2} - 43.0934} \right) - 1.508s_{1} \left( {s_{6}^{2} + s_{7}^{2} } \right)} \\ { + 7.4777\left( {s_{6}^{3} + s_{7}^{3} } \right) + 0.7854\left( {s_{4} s_{6}^{2} + s_{5} s_{7}^{2} } \right)\# \left( {31} \right)} \\ \end{array}$$

Subject to

$$\begin{aligned} & G_{1} = \frac{27}{{S_{1} S_{2}^{2} S_{3} }} - 1 \le 0;\;G_{2} = \frac{397.5}{{S_{1} S_{2}^{2} S_{3}^{2} }} - 1 \le 0;\;G_{3} = \frac{{1.93s_{4}^{2} }}{{S_{2} S_{6}^{2} S_{3} }} - 1 \le 0\;G_{4} = \frac{{1.93s_{5}^{3} }}{{S_{2} S_{7}^{4} S_{3} }} - 1 \le 0 \\ & G_{5} = \sqrt {\sqrt {\frac{{\left( {\frac{{745s_{4} }}{{s_{2} s_{3} }}} \right)^{2} + 16.9 \times 10^{6} }}{{110s_{6}^{3} }}} - 1 \le 0} \quad G_{5} = \sqrt {\sqrt {\frac{{\left( {\frac{{745s_{4} }}{{s_{2} s_{3} }}} \right)^{2} + 16.9 \times 10^{6} }}{{110s_{6}^{3} }}} - 1 \le 0} \\ & G_{7 } = \frac{{s_{2} s_{3} }}{40.0} - 1 \le 0\;and\;G_{8} = \frac{{5s_{2} }}{{s_{1} }} - 1 \le 0\;and\;G_{9 } = \frac{{s_{1} }}{{12s_{2} }} - 1 \le 0 \\ \end{aligned}$$

The decision variables’ ranges are as follows:

$$\begin{aligned} & 2.6 \le s_{1} \le 3.6; \\ & 0.7 \le s_{2} \le 0.8; \\ & 17 \le s_{3} \le 28; \\ & 7.8 \le s_{4} ; \\ & s_{5} \le 8.3; \\ & 2.9 \le s_{6} \le 3.9; \\ & 5 \le s_{7} \le 5.5 \\ \end{aligned}$$

The suggested EOBAVO approach outperformed the selected MAs in determining the ideal cost for the speed reducer design problem, according to the simulation results shown in Table 22.

**Table 22:** EOBAVO’s speed reducer results.

Algorithm	Optimal values of variables							Optimum cost
	${s}_{1}$	${s}_{2}$	${s}_{3}$	${s}_{4}$	${s}_{5}$	${s}_{6}$	${s}_{7}$	
EOBAVO	2.796E+00	7.286E−01	1.964E+01	8.474E+00	2.203E+00	2.921E+00	5.257E+00	***2.686E*+*03***
ROBAVO	3.542E+00	7.026E−01	1.991E+01	8.496E+00	3.558E+00	3.400E+00	5.081E+00	2.995E+03
OBAVO	3.178E+00	7.046E−01	2.532E+01	8.790E+00	7.162E+00	3.306E+00	5.058E+00	3.890E+03
AVO	3.472E+00	7.967E−01	2.741E+01	8.669E+00	2.212E+00	3.621E+00	5.282E+00	5.660E+03
PSO	2.878E+00	7.175E−01	2.717E+01	8.148E+00	2.778E+00	2.957E+00	5.003E+00	3.920E+03
SSA	2.990E+00	7.730E−01	2.136E+01	8.162E+00	3.949E+00	2.961E+00	5.500E+00	3.502E+03
SCA	3.217E+00	7.765E−01	1.912E+01	8.294E+00	1.600E+00	3.155E+00	5.483E+00	3.184E+03
WOA	2.967E+00	7.749E−01	2.097E+01	8.705E+00	7.862E+00	3.760E+00	5.112E+00	3.480E+03

### The Three-bar truss design problems

**Fig. 27:**
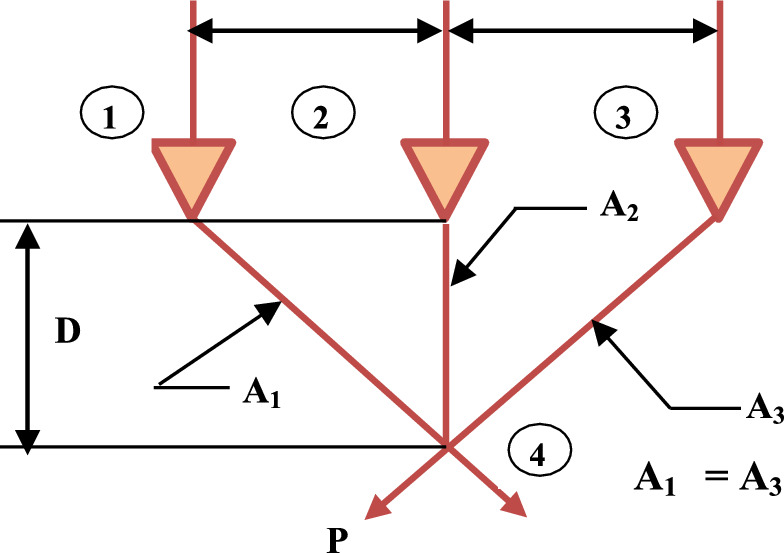
Three bar truss design problems.

The objective of this engineering design is to ascertain the ideal values for two variables, A_1_ and A_2_, to minimize the truss’s weight, subject to three optimization constraints, namely: Defection, stress, and buckling. (a third value, A_3_ = A_1_, is observed). Figure 27 shows the three-bar truss design’s structural outline.

Mathematically, the design’s formula is shown in Eq. (32) as follows:

Consider $$\vec{z} = \left[ {z_{1} z_{2} } \right] = \left[ {A_{1} A_{2} } \right]$$

32 $$\begin{array}{*{20}c} {Minimize f\left( {\vec{z}} \right) = 2\sqrt 2 z_{1} + z_{2} \times l} \\ \end{array}$$

Subject to

$$g_{1} \left( {\vec{z}} \right) = \frac{{\sqrt 2 z_{1} + z_{2} }}{{\sqrt 2 z_{1}^{2} + 2z_{1} z_{2} }}P - \sigma \le 0;\quad g_{2} \left( {\vec{z}} \right) = \frac{{ z_{2} }}{{\sqrt 2 z_{1}^{2} + 2z_{1} z_{2} }}P - \sigma \le 0;$$

$$g_{3} \left( {\vec{z}} \right) = \frac{1}{{\sqrt 2 z_{2} + z_{1} }}P - \sigma \le 0;\quad l = 100\;{\text{cm}},P = 2k\frac{N}{{cm^{3} }},\sigma = \frac{2kN}{{cm^{3} }};$$

The decision variables’ ranges are as follows: $0 \le z_{1} , z_{2} \le 1$.

According to the results shown in Table 23, the recommended EOBAVO technique performs better than other MAs in determining the least cost design value.

**Table 23:** Results of the three-bar truss issue used in EOBAVO.

Algorithm	Optimal variables		Optimum cost
	${A}_{1}$	${A}_{2}$	
EOBAVO	1.189E−01	4.685E−01	***8.048E*+*01***
ROBAVO	5.186E−01	9.657E−01	2.433E+02
OBAVO	4.004E−01	3.625E−01	1.495E+02
AVO	6.076E−01	8.209E−01	2.539E+02
PSO	7.643E−01	1.024E−01	2.264E+02
SSA	8.807E−01	8.867E−01	3.378E+02
SCA	2.616E−01	1.584E−01	8.983E+01
WOA	5.567E−01	6.299E−01	2.204E+02

### Statistical analysis for engineering problems

Statistical analysis is crucial in optimization engineering for objectively evaluating algorithm performance, comparing solutions, and ensuring results are reliable and significant across multiple runs. This also supports parameter tuning, convergence assessment, and robustness evaluation, enhancing the validity and applicability of optimization solutions^75^. Table 24 highlights the comparative performance of EOBAVO and its base version (AVO) and other state-of-the-art MA across sic standard engineering design problems namely Pressure Vessel Design (PVD), Welded Beam Design (WBD), Tension/Compression Spring Design (TCSD), Gear Train Design (GTD), Speed Reducer Design (SRD), Three-Bar Truss Design (TBTD).In general, EOBAVO achieved the best average solution in four out of six design problems and ties for the best in one, demonstrating its superior global search capability. EOBAVO consistently yields the lowest standard deviation in five out of six problems, reflecting remarkable robustness and stability across independent runs. The only exception is the Tension/Compression Spring Design (TCSD) problem, where the Whale Optimization Algorithm (WOA) shows superior consistency despite matching EOBAVO’s average solution. Overall, these results affirm the strength of the opposition-based learning enhancements in improving the exploration–exploitation balance of AVO, enabling it to outperform both its baseline and competing metaheuristic algorithms in complex constrained optimization contexts.

**Table 24:** Comparative performance of EOBAVO and optimizers.

		EOBAVO	AVO	PSO	SSA	SCA	WOA
PVD	AVG	**9.850E+00**	1.047E+01	1.094E+01	1.072E+01	1.090E+01	1.106E+01
	STD	**4.500E−03**	8.000E−03	1.100E−02	9.500E−03	1.050E−02	1.150E−02
WBD	AVG	**7.410E−02**	7.910E−02	8.480E−02	8.210E−02	8.360E−02	8.500E−02
	STD	**1.272E−01**	1.307E−01	1.337E−01	1.322E−01	1.332E−01	1.342E−01
TCSD	AVG	**5.420E−03**	5.880E−03	6.070E−03	**5.420E−03**	6.140E−03	**5.420E−03**
	STD	4.523E−04	8.823E−04	7.223E−04	8.523E−04	1.002E−03	**2.341E−06**
GTD	AVG	**1.989E−02**	**1.989E−02**	2.091E−02	2.075E−02	2.102E−02	2.120E−02
	STD	2.991E−03	4.701E−03	5.769E−03	5.043E−03	5.556E−03	6.197E−03
SRD	AVG	**1.712E+03**	1.757E+03	1.832E+03	1.805E+03	1.840E+03	1.863E+03
	STD	**5.000E+00**	7.500E+00	9.800E+00	8.500E+00	9.300E+00	1.050E+01
TBTD	AVG	**1.184E−01**	1.243E−01	1.297E−01	1.276E−01	1.292E−01	1.308E−01
	STD	**5.500E−02**	8.200E−02	1.150E−01	1.000E−01	1.100E−01	1.250E−01

**Fig. 28:**
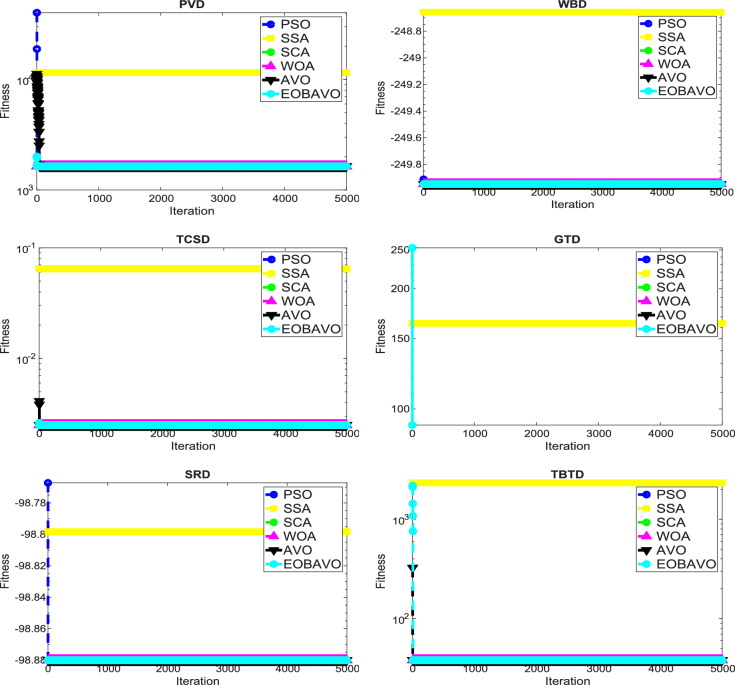
Convergence curves of EOBAVO and other Optimizers on Engineering design problems.

Figure 28 presents the convergence curves of six algorithms—PSO, SSA, SCA, WOA, AVO, and EOBAVO—across six engineering design problems (PVD, WBD, TCSD, GTD, SRD, and TBTD). The EOBAVO consistently achieves superior fitness values across all test functions, converging more rapidly and attaining lower objective function values than the competing algorithms. Notably, in PVD and TBTD, EOBAVO demonstrates faster convergence toward the global optimum while maintaining stability, outperforming AVO and the other baseline algorithms. In contrast, SSA exhibits poor convergence across most functions and yielding suboptimal fitness values, particularly evident in PVD and TCSD. PSO and SCA display moderate convergence but plateau prematurely without significant improvement over iterations. WOA shows competitive performance in some cases but fails to match EOBAVO’s convergence precision. Overall, the results validate that integrating opposition-based learning into AVO (i.e., EOBAVO) enhances both convergence speed and solution accuracy, confirming its robustness and effectiveness in solving these benchmark optimization problems.

### Utilizing the EOBAVO algorithm to address the issue of wind turbine installation

The electrical energy generated when wind is converted into mechanical energy by windmills or turbines is known as Wind energy. This is one of the most significant renewable energy bases because of its widespread availability and abundance. The effective utilization of this energy has the potential to produce a remarkable amount of electricity. Wind energy has recently become more popular due to the rise in the need for electricity. Strategically positioning the wind turbines in a wind farm can lead to increased total electricity production. The wake effect may cause most wind farms to produce less electricity, and the haphazard placement of the turbines within wind farms can hinder turbines from reaching their full capacity. Effective management of the wake effect, combined with the strategic positioning of wind turbines, can lead to an increase in the energy yield from wind farms. Optimizing the output of these facilities is vital for lowering energy expenditures. In this section, the recommended EOBAVO technique is employed to ascertain the most advantageous sites for wind turbines, ensuring that the highest power output is achieved at the lowest cost per kilowatt.

The research encompasses two separate case studies: The first examines Constant Wind Speed (CWS) in conjunction with Variable Wind Direction (VWD), while the second investigates Variable Wind Speed (VWS) paired with Variable Wind Direction (VWD). In comparison to the statistical results, LSHADE^32^ , GWO^8^, GA^76^, GA^77^, RSA^78^, SBO^79^, and BPSO TVAC^80^ are used.

**Fig. 29:**
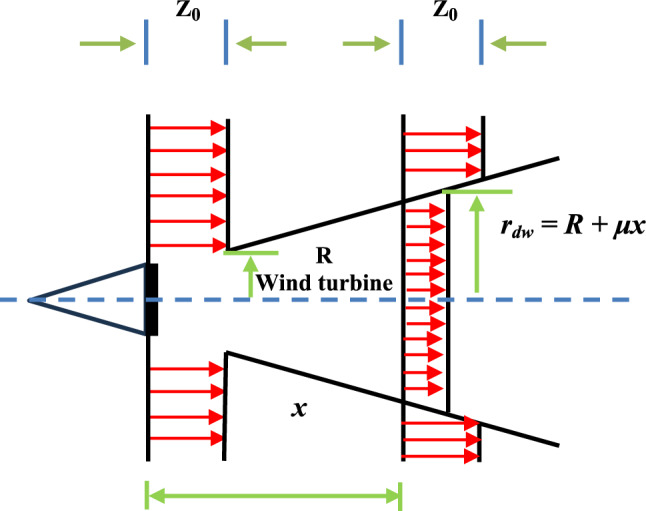
Jensen Wake effect model (Schematic).

The nearby turbines in wind turbine fields have an impact on the wind’s speed. A wake forms downstream of a wind turbine when it uses the wind to generate power. The downstream turbine’s power output decreases compared to the turbine running in a free wind flow if the nearby turbine is operating within the wake. A module developed by N.O. Jensen, known as the wake effect model, was used in the majority of the wind turbine layout optimization studies^44,81^. This model is shown in Fig. 29.

At a downstream distance of x from the wind turbine, the wind speed v in the wake is represented using Eqs. (33)–(36) as follows:

33 $$\begin{array}{*{20}c} {v = v_{0} \left[ {1 - \frac{2a}{{\left( {1 + \mu \left( \frac{x}{rdr} \right)} \right)^{2} }}} \right]} \\ \end{array}$$

34 $$\begin{array}{*{20}c} {rdr = r_{r} \sqrt {\frac{1 - a}{{1 - 2a}}} } \\ \end{array}$$

35 $$\begin{array}{*{20}c} {C_{t} = 4a\left( {1 - a} \right)} \\ \end{array}$$

36 $$\begin{array}{*{20}c} {\mu = \frac{0.5}{{ln\left( {\frac{h}{{h_{0} }}} \right)}}} \\ \end{array}$$

where μ is the entrainment constant, *v*_*0*_ is the velocity of the free stream, and ***a*** is the induction factor for axial. The radius of the downstream rotor is represented by *rdr*, the wind turbine’s thrust coefficient by *C*_*t*_, the rotor radius by *r*_*r*_, the hub height by h, and the turbine’s surface roughness by *h*_*0*_.

Using Eq. (37), at any distance *x*, the downstream wake radius (*r*_*dw*_) is estimated.

37 $$\begin{array}{*{20}c} {r_{dw} = R + \mu x} \\ \end{array}$$

where the wind turbine radius is indicated by R.

The total of the kinetic energy defects is the mixed wake kinetic energy when one turbine passes next to multiple turbines. Thus, the downstream velocity of the N turbine is calculated using Eq. (38).

38 $$\begin{array}{*{20}c} {\left( {1 - \frac{v}{{v_{0} }}} \right)^{2} = \mathop \sum \limits_{i = 1}^{N} \left( {1 - \frac{{v_{i} }}{{v_{0} }}} \right)^{2} } \\ \end{array}$$

Every wind turbine produces power and the sum is determined by Eq. (39):

39 $$\begin{array}{*{20}c} {P_{wt} = \mathop \sum \limits_{i = 1}^{N} 0.3 \times v^{3} } \\ \end{array}$$

The wind farm’s wind turbines serve as the basis for the cost model created by^76^. According to this model expression, establishing a single wind turbine cost is 1, whereas adding many wind turbines can save costs by one-third. The remaining expenses require two thirds. The wind farm costs are computed using the expression shown in Eq. (40)

40 $$\begin{array}{*{20}c} {Cost = N\left[ {\frac{2}{3} + \frac{1}{3}e^{{ - 0.00174N^{2} }} } \right]} \\ \end{array}$$

The objective of the cost function is to reduce the investment cost associated with each unit of energy generated to the minimum, while simultaneously enhancing the power output of a wind farm. The objective function is then shown in Eq. (41) as follows:

41 $$\begin{array}{*{20}c} {Objective\;function = \frac{Cost}{{P_{wt} }}} \\ \end{array}$$

**Fig. 30:**
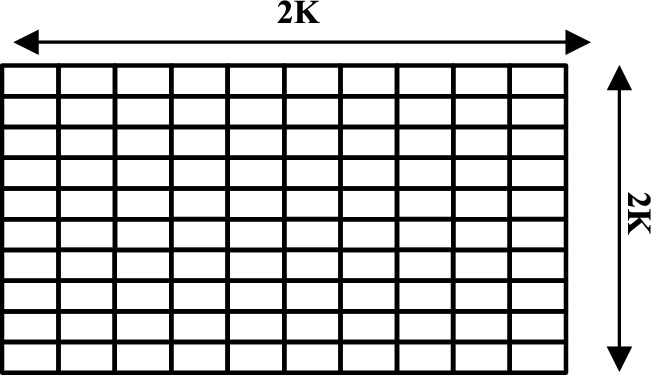
Wind farm area subdivision.

For wind turbine’s optimal situation in the field, the 2000 × 2000 m^2^ farm area is partitioned into 10 × 10 square grids. This particular arrangement of grids permits the construction of 100 turbines in a wind farm. A wind turbine (1) or nothing (0) can be placed in every grid cell of the wind farm. As shown in Fig. 30, the width of a cell is five times that of a rotor diameter, which requires a rotor diameter of 200 m for horizontal directions and 40 m for vertical directions.

Table 25 shows the characteristics of wind turbines. The findings are evaluated against other cutting-edge algorithms such as LSHADE^32^, GWO^8^, GA^76^, GA^77^, RSA^78^, SBO^79^, and BPSO TVAC^80^. The suggested EOBAVO approach is combined with CWS with VWD and VWS with VWD. It should be mentioned that each algorithm has a maximum of 100 iterations and 200 populations. Additionally, the upper and lower bounds are 0 and 1, and the problem size is 100.

**Table 25:** Specifications of wind turbine.

Parameters	Value
Height of hub (*h*)	60 m
Rotor diameter (*2r*_*dr*_)	40 m
Surface roughness of wind turbine (*h*_*0*_)	0.3 m
Thrust coefficient (*C*_*t*_)	0.88

Two case studies are examined below to assess the efficacy of the recommended strategy for the optimal placement of a wind turbine:

#### VWD combined with CWS

**Fig. 31:**
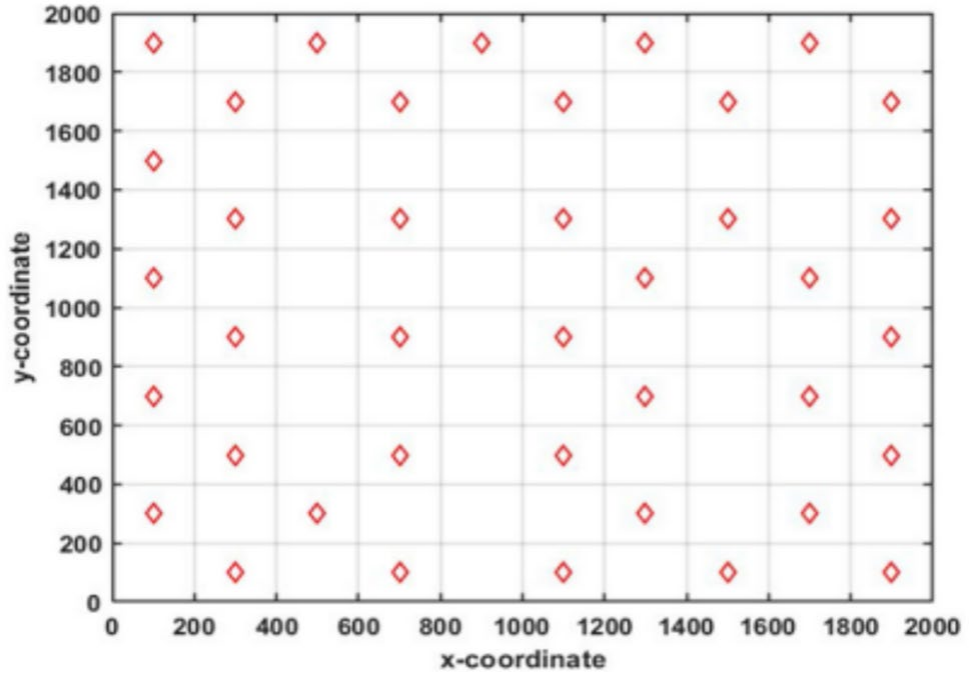
EOBAVO’s wind farm setup for a 10 × 10 square grid for CWS with VWD.

At this instance, the wind moves in different directions and surrounding the wind farm increases at equal interval of 10° between 0° to 360°, with a fixed speed of 12 m/s. The statistical results for different MAs and the suggested EOBAVO approach are given in Table 26. The table makes it clear that EOBAVO method performs better than the other MAs. Additionally, the optimal wind turbine location in a wind farm as established by the EOBAVO approach is shown in Fig. 31. The EOBAVO technique uses 40 turbines to produce 18,120 kW at an efficiency of 88.31% and a cost of 0.001734/kW.

**Table 26:** Results of comparing VWD with CWS.

Algorithm	No. of turbines	Cost/kW	Power (kW)	Efficiency
EOBAVO	40	1.73E−03	18,120	88.31%
LSHADE^32^	40	1.73E−03	18,117	88.31%
GWO^8^	40	1.74E−03	18,018	87.91%
GA^76^	19	1.94E−03	9,448	87.61%
GA^77^	39	1.77E−03	17,417	95.31%
SBO^79^	40	1.79E−03	17,452	86.61%
RSA^78^	39	1.75E−03	17,607	NA
BPSO−TVAC^80^	35	1.76E−03	15,998	88.41%

#### VWD combined with VWS

**Fig. 32:**
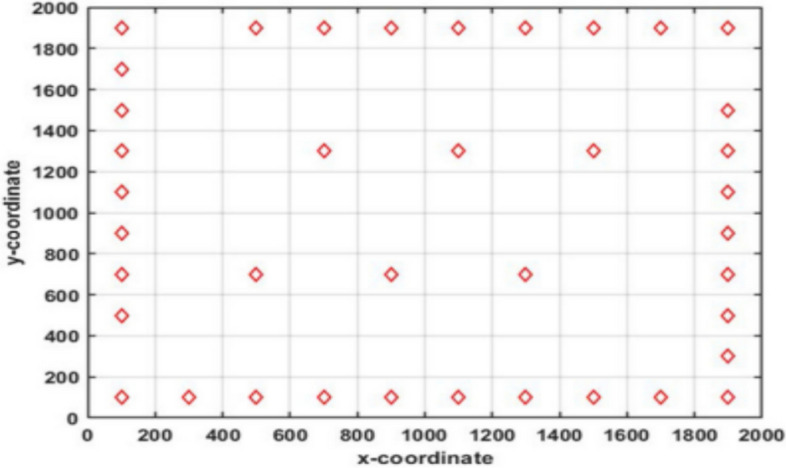
EOBAVO’s wind farm setup for a 10 × 10 square grid for VWS with VWD.

In this instance, the wind flows around the wind farm in three different directions at speeds of 8, 12, and 17 m/s. It also increases the angles by 100°, ranging from 0° to 360°. The statistical results of the various MAs and the proposed EOBAVO approach are shown in Table 27. The table makes it clear that the EOBAVO approach fared better than the other metaheuristic algorithms. Additionally, the optimal wind turbine location in a wind farm, as established by the EOBAVO approach, is shown in Fig. 32. The EOBAVO uses 39 turbines to produce 30,830 kW at an efficiency of 84.49% and a cost of 0.000807/kW.

**Table 27:** Results of comparing VWD with VWS.

Algorithm	No. of turbines	Cost/kW	Power (kW)	Efficiency
EOBAVO	39	**0.000807**	30,830	84.49%
LSHADE ^32^	39	0.000807	30,829	84.49%
GWO^8^	39	0.000827	34,911	84.19%
GA^76^	46	0.000969	11,935	83.89%
GA^77^	15	0.000817	30,331	91.59%
SBO^79^	40	0.000824	30,982	82.89%
RSA^78^	39	0.000816	30,575	NA
BPSO-TVAC^80^	38	0.000812	14,277	84.69%

Finally, the results clearly show that the EOBAVO technique performed better than other algorithms in terms of setting up the wind turbines in both case studies. Finally, it can be said that the suggested EOBAVO has outperformed the other selected algorithms on a range of practical engineering design and applications issues.

## Conclusion

OBL, which has gained increased attention recently, is used to improve the efficacy of metaheuristic algorithms. To achieve varying densities across all vulture species and to foster population diversity, this research intends to refine the recently developed nature-inspired algorithm known as the African Vulture Optimizer (AVO). This enhancement will involve the incorporation of an opposition-based learning mechanism, resulting in the creation of the Opposition-Based African Vulture Optimizer Algorithm (OBAVO). Nevertheless, this approach elevates the computational demands necessary for discovering superior solutions and is susceptible to premature convergence. To effectively resolve these issues while steering clear of local optima and facilitating faster convergence, a novel adaptation of Opposition-based Learning, called Evolved Opposition-based Learning (EOBL), has been proposed. This adaptation is incorporated into a refined version of the African Vulture Optimizer Algorithm, known as the Enhanced Opposition-based African Vulture Optimizer Algorithm (EOBAVO). The investigation’s findings demonstrate that, for a significant number of the functions studied, both the accuracy of the solutions and the convergence rates were notably superior when assessed against other optimization techniques. Evaluations of engineering difficulties show that the newly created EOBAVO performs better than its contemporaries. In contrast to other Metaheuristics, this one is negligible when it comes to solving multi-modal functions. While the proposed EOBAVO algorithm demonstrates strong performance across a wide range of optimization tasks, future work will focus on addressing its limitations on highly ill-conditioned and rotated problems (observed in CEC2022 functions CEC01–CEC03). Incorporating hybrid local search technique and developing rotation-invariant opposition strategies are promising directions to further enhance EOBAVO’s adaptability and performance in complex, non-separable optimization landscapes. Future initiatives designed to minimize the frequency of function calls are projected to find utility in several domains, such as image processing, feature selection, and data mining. We also aim to extend the proposed EOBAVO framework to handle discrete and mixed-integer optimization problems in our future works, which are prevalent in engineering design. This adaption will involve redefining OBL mechanisms for discrete spaces, hybrid encoding strategies for mixed variables, and specialized constraint-handling techniques to maintain feasibility while preserving diversity and convergence speed.

## Data Availability

The datasets used and/or analysed during the current study available from the corresponding author on reasonable request.
